# Sepsis and septic shock in France: incidences, outcomes and costs of care

**DOI:** 10.1186/s13613-020-00760-x

**Published:** 2020-10-20

**Authors:** Claire Dupuis, Lila Bouadma, Stéphane Ruckly, Anne Perozziello, Damien Van-Gysel, Arthur Mageau, Bruno Mourvillier, Etienne de Montmollin, Sébastien Bailly, Gregory Papin, Fabrice Sinnah, Camille Vinclair, Sonia Abid, Romain Sonneville, Jean-François Timsit

**Affiliations:** 1 Medical Intensive Care Unit, Gabriel Montpied University Hospital, Clermont-Ferrand, France; 2 UMR 1137-IAME Team 5–DeSCID: Decision Sciences in Infectious Diseases control and care INSERM/Univ Paris Diderot, Sorbonne Paris Cité, 75018 Paris, France; 3 Medical and Infectious Diseases Intensive Care Unit, Bichat Claude Bernard University Hospital, AP-HP, 46 rue Henri Huchard, 75018 Paris, France; 4 Département D’Informations Médicales, Bichat Claude Bernard University Hospital, AP-HP, 46 rue Henri Huchard, 75018 Paris, France; 5 Medical Intensive Care Unit, Robert Debré University Hospital, Rue du Général Koenig, 51100 Reims, France; 6 HP2 Laboratory, INSERM U1042, Univ. Grenoble Alpes, Grenoble, France; 7 EFCR Laboratory, Grenoble Alpes University Hospital, Grenoble, France; 8 Department of Anaesthesia and Intensive Care, Bichat Claude Bernard University Hospital, AP-HP, 46 rue Henri Huchard, 75018 Paris, France; 9 Intensive Care Unit, Centre Hospitalier de La Côte Basque, 64109 Bayonne, France

**Keywords:** Sepsis, Septic shock, Epidemiology, Secular trends

## Abstract

**Background:**

Sepsis is one of the leading causes of death worldwide. The associated incidence, mortality and trends do not differ greatly between documented reports. The purpose of this study was to provide an in-depth description of patients with sepsis and septic shock hospitalized in France from 2010 to 2015 and to explore the temporal trends of their clinical characteristics, costs and outcomes.

**Methods:**

Retrospective cohort study of the French hospital administrative database in which organ failure therapies and severity scores are systematically registered. All patients admitted between 2010 and 2015 for sepsis and septic shock as defined by an ICD-10 code for infection, and for organ failure or the use of organ failure supplementation were included. Incidence, outcomes and trends were analyzed. Subgroup analyses based on several coding strategies and adjusted for severity scores were performed.

**Results:**

A total of 737,147 patients with sepsis and 492,902 patients with septic shock were included. From 2010 to 2015, the incidence of sepsis and septic shock increased, respectively, from 206 to 243 and from 135 to 171 cases per 100,000 population. Case fatality remained at 34% for sepsis, but decreased from 46 to 44% for septic shock.

Median hospital stay costs amounted to €11,400 (IQR: 5036; 24,364) for patients with sepsis and €16,439 (IQR: 7339; 29,360) for patients with septic shock.

After adjustment for case-mix and illness severity, the risk of death was stable for sepsis (0.08% [− 0.04; 0.20] per year), but decreased for sepsis patients admitted to the intensive care unit and for cases of septic shock (− 0.33%[ − 0.40; − 0.27] per year).

**Conclusions:**

Sepsis is common, frequently fatal and expensive to treat. Its incidence has increased. Case fatality has decreased in most severely affected patients, owing partly to general improvements in care.

## Background

Sepsis is one of the leading causes of death worldwide and is a major public health issue [1–5]. The Intensive Care Over Nations (ICON) Audit [1] showed that sepsis accounts for 30% of reasons for intensive care unit (ICU) admission and is associated with a hospital mortality rate of 35%. A recent review of 27 epidemiologic studies, mostly based on North American, Australian and North European administrative databases [2], showed an incidence rate of sepsis cases of 270 per 100,000 person-years (95% CI, 176–412) and an in-hospital mortality rate of 26%. However, previous results extracted from administrative databases were quite heterogeneous, mostly because of the differences in the International Classification of Diseases (ICD) coding strategies used to define sepsis [6, 7] (Additional file 1: Table S1).

In France, the medico-economic administrative hospital database called PMSI (French acronym for “Programme de Médicalisation des Systèmes d'Information”) provides data on all hospital stays in France. Unlike other systems, the PMSI includes mandatory calculations of the Simplified Acute Physiology Score II (SAPS II) severity scores and daily monitoring of organ failure supplementation.

Our aim was to provide an up-to-date report on the epidemiology and management of sepsis and septic shock in France between 2010 and 2015, and to explore the temporal trends of their clinical characteristics, costs and outcomes.

## Methods

### Study design and setting

We analyzed all hospital stays in France from January 1, 2010 to December 31, 2015 of patients older than 18 years identified by codes corresponding to sepsis or septic shock.

### Data source

The French healthcare system covers both public and private hospitals which provide healthcare to every resident in the country. Most French healthcare costs are covered by a public healthcare insurance scheme. Data on all hospital stays are thus recorded in the PMSI.

Demographic and clinical data were collected from the PMSI database including all discharge diagnoses (ICD-10 codes [International Classification of Diseases, Tenth Revision)]), daily records of medical procedures performed during hospital stays (CCAM codes [French acronyms for “Classification Commune des Actes Médicaux”]) including organ failure supplementation, date of discharge, length of stay(LOS), diagnosis-related groups (GHM [French acronym for ‘‘Groupe Homogène de Malades’’]) to classify patients into subgroups according to medical procedures and discharge diagnoses, and finally SAPS II score in case of ICU admission. We estimated the direct cost of each hospital stay. Briefly, it is the sum of a global price defined by the main diagnosis of the hospital stay and severity of the disease and some supplements based on length of stay and special procedures performed during hospital stays such as renal replacement therapy. More details are given in the online daily supplement (Additional file 1: Fig. S1). The estimates of the yearly French total population were provided by the French National Institute for Statistics and Economic Studies, a publicly available database of census records for the entire country [8].

### Definitions

**Fig. 1:**
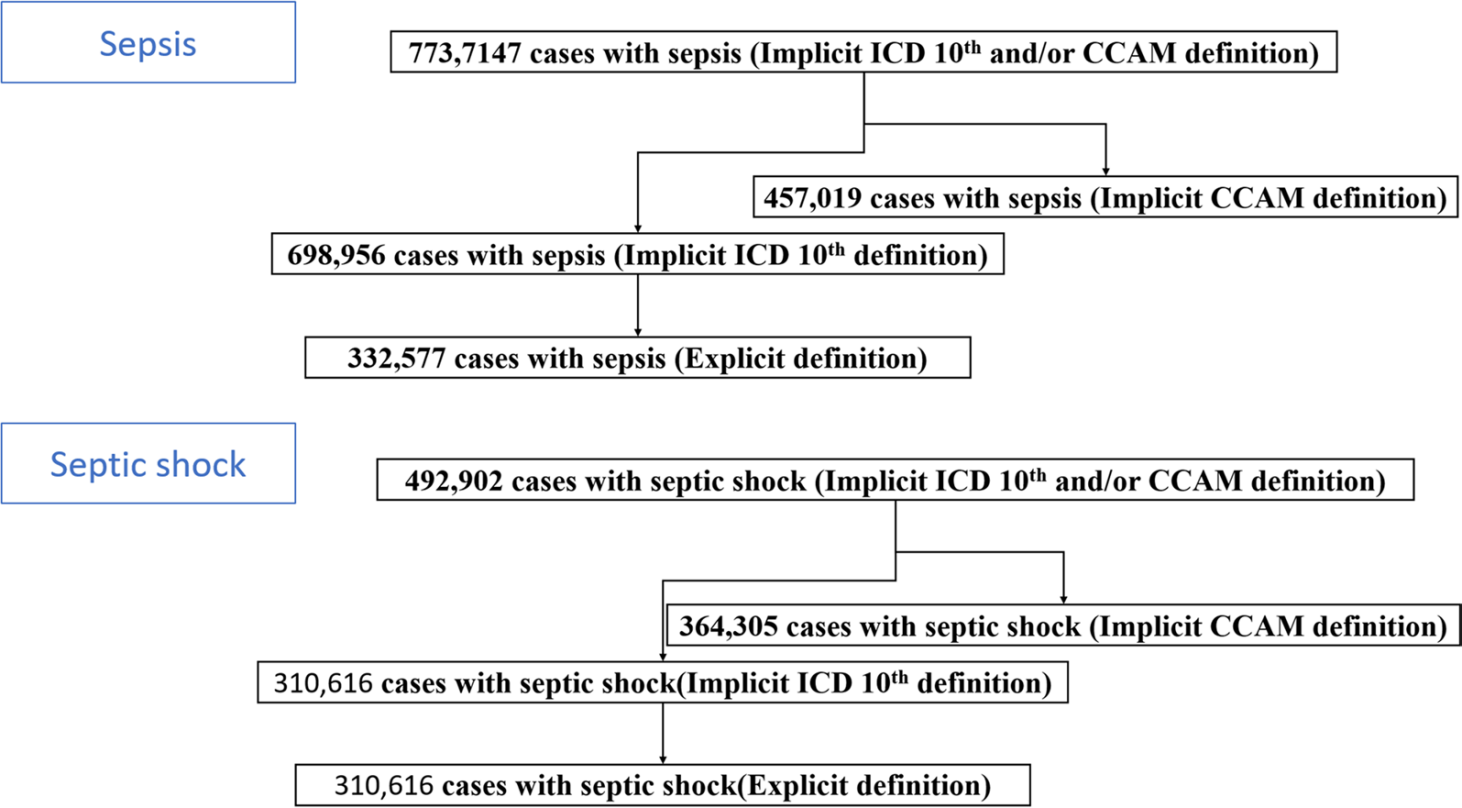
ICD-10: International Statistical Classification of Diseases, 10th Edition. CCAM: “classification commune des actes médicaux”. Implicit definition of sepsis: a code for infection using ICD-10 and a code for organ failure and/or a procedure code for the use of organ failure supplementation (using ICD-10 and/or CCAM). Explicit definition of sepsis: ICD-10 codes including R651 or R572 or R578 or R579. Implicit definition of septic shock: a code for infection using ICD-10 and a code for shock and/or a procedure code for the use of vasopressor (using ICD-10 and/or CCAM). Explicit definition of septic shock: ICD-10 codes including R572 or R578 or R579

Codes attributed during all hospital stays were considered. Among adults older than 18 years old, hospital stays for sepsis and septic shock were identified by ICD-10 and/or procedure codes according to explicit definitions using the direct codes for sepsis and septic shock (Fig. 1) [6, 7] or according to implicit definitions with an ICD-10 code for infection and a diagnostic code for organ failure for sepsis (or a diagnostic code for shock for septic shock) or a procedure code for the use of organ failure supplementation for sepsis (or of only vasopressor for septic shock). Several implicit definitions were compared depending on the infection codes (a wide definition including more than 1,000 codes, similar to that used by Fleischman [7], and a narrower definition only focusing on septicemia codes [9]), and on the definitions of organ failure (a. procedure codes or ICD-10 codes; b. ICD-10 codes; c. procedure codes) (Additional file 1: Tables S2, S3).

### Characteristics recorded

A hospital stay was analyzed from admission to death or discharge, taking into account all potential transfers. For each hospital stay, the following data were obtained: demographics, hospital characteristics, reason for admission, readmissions, wards where the patients were admitted, patient comorbidities according to ICD-10 Charlson score [10], severity of disease on admission (organ failures and SAPS II only for patients admitted to the ICU) and the characteristics of the sepsis (Additional file 1: Tables S2, S3). The direct costs of each hospital stay were estimated. ICU and hospital LOS, and outcomes including death and discharge home were recorded.

### Aim of the study

The aim of our study was to explore the temporal trends in clinical characteristics, case-mix, costs and outcomes of septic and septic shock patients in France from 2010 to 2015.

### Sensitivity and subgroup analyses

Subgroup analyses were performed according to the definitions of sepsis and septic shock, but also among patients admitted to the ICU and those never admitted.

### Statistical analyses

First, standard descriptive statistics were performed for the whole cohort and by year from 2010 to 2015. Data were summarized as frequency and percentage for categorical variables, and median and interquartile for continuous variables. Second, the incidence and hospital mortality rates were estimated from national population data expressing the results per 100,000 inhabitants. The in-hospital case-fatality rates (CFR) were calculated as the number of in-hospital deaths divided by the number of cases, expressed as percentages. The age-adjusted rates (incidence, mortality) were calculated by direct standardization based on the size of the population living in France in 2010 [7]. Age and CFR were calculated by direct standardization based on the 2010 data [7]. Third, the temporal trends of all the variables of interest were assessed. Cochran Armitage tests of trends and linear trend analyses to explore any changes from 2010 to 2015 were conducted. Fourth, multivariate hierarchical logistic regression analyses with a random center effect were performed to identify the factors associated with death. The variables were selected by univariate analyses with a threshold of 0.1, after which a backward selection was performed in the multivariate analyses. The tested covariates are listed in the supplementary data. Of these covariates, the SAPS II score was only assessed in the subgroup of the ICU-admitted patients. The covariates ‘Year of admission’ and ‘Readmissions during the year following the last admission’ were forced into each model. All these analyses were performed in each of the pre-determined subgroups. P-values less than 0.05 were considered significant. Fifth, from the adjusted odds ratio and the prevalence of death in the whole cohort of patients with sepsis or septic shock, we determined the relative risk of death of each year relative to that of 2010 [11]. Finally, the adjusted increased risk of death per year was derived from the slope of the regression line through the adjusted relative risk of death per year. Analyses were performed solely of characteristics and cases identified according to our coding strategies. Data were analyzed with SAS® (Version 9.4; SAS Institute, Cary, NC, USA) and R (Version 3.4.0; R Core Team, Wien, Austria).

### Ethical aspects

In accordance with the French regulatory system regarding personal and medical data and after agreement of the French data protection authority (CNIL), our institution was allowed to access the PMSI database. We only had access to patients diagnosed with sepsis or septic shock according to our definition, and to pseudonymized data.

## Results

### Incidence

**Fig. 2.**
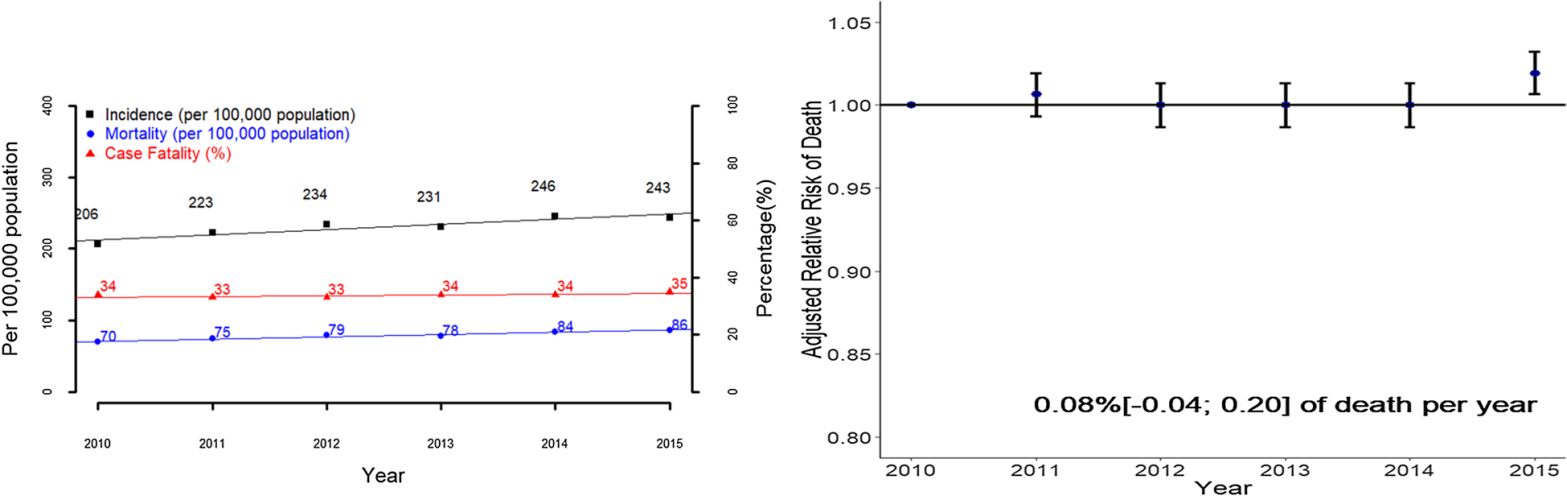
Incidence, mortality, case fatality from 2010 to 2015 in the whole cohort of patients with sepsis (**a**) and adjusted relative risk of hospital death rate from 2010 to 2015, in the whole cohort of patients with sepsis (**b**). The adjusted relative risk of hospital death was based on the adjusted odd ratio of death obtained by a multivariate hierarchical logistic regression analysis with a random center effect taking into account all the associated factors of hospital death (Additional file 1: Table S11)

From 2010 to 2015, a total of 737,147 sepsis hospital stays from 1431 hospitals were recorded, with an implicit definition based on ICD-10 and/or CCAM coding, including 332,577 cases with an explicit definition, 519,049 cases admitted to the CU, and 141,669 cases with a code for septicemia (Fig. 1, Additional file 1: Table S4). The standardized incidence of sepsis defined implicitly increased significantly in France from 206 per 100,000 inhabitants in 2010 to 243 per 100,000 inhabitants in 2015 (Fig. 2, Table 1). Similar trends were observed with regard to sepsis implicitly defined by the ICD-10 code and CCAM codes or explicitly defined, and among patients admitted to the ICU and those not admitted but not in the subgroup of patients with septicemia (Additional file 1: Fig S2, Tables S5–S7).

**Table 1:** Main characteristics of the included sepsis hospital stays in France between 2010 and 2015 and their associated temporal trends

Sepsis Implicit definition ICD 10th or CCAM	All	2010	2011	2012	2013	2014	2015	Trends^a^
Cases	737,147	105,733	116,139	123,792	123,950	133,651	133,882	
Incidence per 100.000 (age-standardized)		206	223	234	231	246	243	↗
Deaths per 100.000 (age-standardized)		70	75	79	78	84	86	↗
Case fatality (age standardized)		34	33	33	34	34	35	=
Hospital stay characteristics								
Services of admission								
Intensive care unit	388,057 (52.6)	56,314 (53.3)	60,150 (51.8)	63,662 (51.4)	64,436 (52)	70,743 (52.9)	72,752 (54.3)	↗
Intermediate care facilities	198,626 (26.9)	24,112 (22.8)	28,671 (24.7)	32,556 (26.3)	34,917 (28.2)	38,780 (29)	39,590 (29.6)	↗
Characteristics of the patients								
Baseline characteristics								
Age in years, median (IQR)	71 [59; 81]	70 [58; 80]	71 [58; 80]	71 [59; 81]	71 [59; 81]	71 [60; 81]	71 [60; 81]	↗
Gender (female)	287,652 (39)	41,353 (39.1)	45,511 (39.2)	48,465 (39.2)	48,144 (38.8)	52,033 (38.9)	52,146 (38.9)	=
Comorbidities								
Myocardial infarction	134,179 (18.2)	19,209 (18.2)	20,933 (18)	22,639 (18.3)	22,539 (18.2)	24,538 (18.4)	24,321 (18.2)	=
Congestive heart failure	222,310 (30.2)	30,211 (28.6)	33,946 (29.2)	37,440 (30.2)	37,837 (30.5)	41,556 (31.1)	41,320 (30.9)	↗
Dementia	36,808 (5)	5209 (4.9)	5943 (5.1)	6416 (5.2)	6071 (4.9)	6722 (5)	6447 (4.8)	↘
Chronic pulmonary disease	135,443 (18.4)	18,608 (17.6)	20,608 (17.7)	22,724 (18.4)	22,514 (18.2)	25,356 (19)	25,633 (19.1)	↗
Diabetes without chronic complication	127,251 (17.3)	17,831 (16.9)	19,051 (16.4)	21,231 (17.2)	21,563 (17.4)	24,185 (18.1)	23,390 (17.5)	↗
Diabetes with chronic complications	73,562 (10)	11,029 (10.4)	11,591 (10)	12,682 (10.2)	11,938 (9.6)	13,432 (10.1)	12,890 (9.6)	↘
Renal disease	105,153 (14.3)	12,925 (12.2)	15,158 (13.1)	17,544 (14.2)	18,002 (14.5)	20,969 (15.7)	20,555 (15.4)	↗
Any malignancy	161,307 (21.9)	22,040 (20.8)	24,530 (21.1)	26,863 (21.7)	27,548 (22.2)	30,434 (22.8)	29,892 (22.3)	↗
Moderate or severe liver disease	56,528 (7.7)	8224 (7.8)	9061 (7.8)	9643 (7.8)	9735 (7.9)	10,132 (7.6)	9733 (7.3)	↘
Metastatic solid tumor	52,933 (7.2)	6420 (6.1)	7616 (6.6)	8558 (6.9)	9128 (7.4)	10,555 (7.9)	10,656 (8)	↗
Charlson score, median (IQR)	2 [1; 4]	2 [1; 4]	2 [1; 4]	2 [1; 4]	2 [1; 4]	2 [1; 5]	2 [1; 5]	↗
Illness severity								
Cardio-vascular organ failure	514,797 (69.8)	70,002 (66.2)	79,693 (68.6)	85,081 (68.7)	86,258 (69.6)	95,238 (71.3)	98,525 (73.6)	↗
Neurological organ failure	184,188 (25)	22,442 (21.2)	26,932 (23.2)	30,623 (24.7)	31,451 (25.4)	35,160 (26.3)	37,580 (28.1)	↗
Renal organ failure	294,273 (39.9)	40,331 (38.1)	44,465 (38.3)	48,585 (39.2)	50,323 (40.6)	54,827 (41)	55,742 (41.6)	↗
Respiratory organ failure	297,664 (40.4)	42,835 (40.5)	46,876 (40.4)	49,722 (40.2)	50,134 (40.4)	53,558 (40.1)	54,539 (40.7)	=
Number of organ failure, median (IQR)	3 [2; 4]	3 [2; 4]	3 [2; 4]	3 [2; 4]	3 [2; 4]	3 [2; 4]	3 [2; 4]	↗
SAPS II score, median (IQR) (miss = 241,497¤)	48 [35; 64]	48 [35; 64]	48 [35; 64]	48 [35; 64]	48 [35; 64]	48 [35; 64]	49 [36; 65]	↗
Outcomes								
Hospital LOS(days) (median [IQR])	17 [7; 34]	17 [7; 35]	17 [7; 34]	17 [7; 34]	17 [7; 34]	17 [8; 34]	17 [7; 33]	↘
ICU LOS(days) (median [IQR]) (missing = 332,463)	8 [3; 17]	8 [3; 19]	8 [3; 18]	8 [3; 17]	8 [3; 17]	7 [3; 17]	7 [3; 16]	↘
Discharge to home	391,409 (53.1)	55,590 (52.6)	62,161 (53.5)	66,413 (53.6)	66,035 (53.3)	71,825 (53.7)	69,385 (51.8)	↘
Hospital mortality	254,013 (34.5)	36,206 (34.2)	39,505 (34)	42,111 (34)	42,415 (34.2)	45,797 (34.3)	47,979 (35.8)	↗
Cost (€), median (IQR)	11,400 [5037; 24363]	11,705.9 [5088; 25364]	10,991.2 [4778; 24209]	10,856.1 [4789; 23810]	11,314 [4938; 24378]	11,648.3 [5164; 24362]	11,847.3 [5218; 24192]	=

^a^Test of trend or linear regression for values from 2010 to 2015; ↗ a significant increase is observed with a *p* value < 0.05; ↘ a significant decrease is observed with a p value < 0.05; ‘ = ’ no significant trend is observed; ¤ Missing values of 241,497 because SAPS II score was only recorded in case of ICU stay; SAPS II: Simplified Acute Physiology Score II; LOS: length of stay; ICU: intensive care unit

**Fig. 3.**
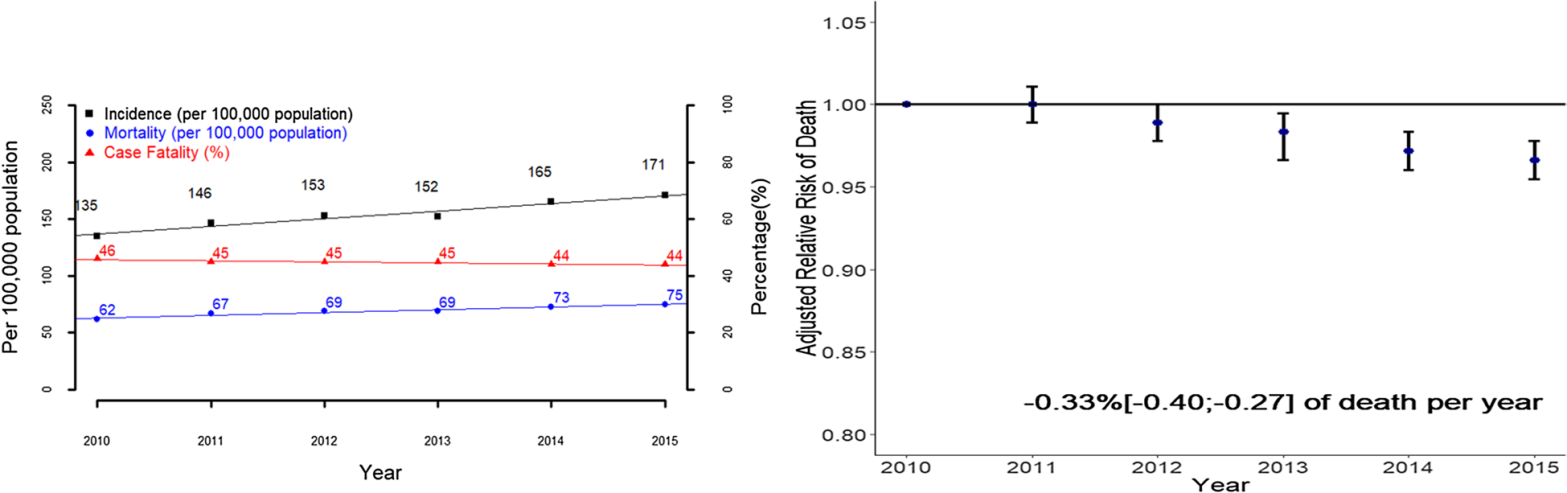
Incidence, mortality, case fatality from 2010 to 2015 in the whole cohort of patients with septic shock (**a**) and adjusted relative risk of hospital death rate from 2010 to 2015, in the whole cohort of patients with septic shock (**b**). The adjusted relative risk of hospital death was based on the adjusted odd ratio of death obtained by a multivariate hierarchical logistic regression analysis with a random center effect taking into account all the associated factors of hospital death (Additional file 1: Table S11)

Similarly, from 2010 to 2015, 492,902 hospital stays due to septic shock with an implicit definition, including 310,616 with an explicit definition, 421,026 admitted to ICU and 113,722 with septicemia were included (Fig. 1). The standardized incidence of septic shock increased significantly from 135 to 171 per 100,000 inhabitants in France from 2010 to 2015 (Fig. 3, Table 2). Similar trends were observed regardless of the septic shock definition used and among patients admitted and not admitted to the ICU, but not in the subgroup of patients with septicemia (Additional file 1: Fig S3, Tables S8–S10).

**Table 2:** Main characteristics of the included septic shock hospital stays in France between 2010 and 2015 and their associated temporal trends

Septic shock Implicit definition ICD 10th or CCAM	All	2010	2011	2012	2013	2014	2015	Trends^a^
Cases	492,902	69,466	76,350	81,107	81,886	89,934	94,159	
Incidence per 100.000 (age-standardized)		135	146	153	152	165	171	↗
Deaths per 100.000 (age-standardized)		62	67	69	69	73	75	↗
Case fatality (age standardized)		46	45	45	45	44	44	↘
Hospital stay characteristics								
Services of admission								
Intensive care unit	353,190 (71.7)	50,369 (72.5)	54,181 (71)	57,553 (71)	58,678 (71.7)	64,696 (71.9)	67,713 (71.9)	=
Intermediate care facilities	151,357 (30.7)	17,917 (25.8)	21,415 (28)	24,475 (30.2)	26,486 (32.3)	29,721 (33)	31,343 (33.3)	↗
Characteristics of the patients								
Baseline characteristics								
Age in years, median (IQR)	70 [59; 80]	70 [58; 79]	70 [58; 80]	70 [59; 80]	70 [59; 80]	70 [59; 80]	70 [60; 80]	↗
Gender (female)	190,941 (38.7)	27,069 (39)	29,513 (38.7)	31,535 (38.9)	31,811 (38.8)	34,677 (38.6)	36,336 (38.6)	=
Comorbidities								
Myocardial infarction	90,838 (18.4)	12,922 (18.6)	13,913 (18.2)	15,084 (18.6)	15,111 (18.5)	16,656 (18.5)	17,152 (18.2)	=
Congestive heart failure	147,098 (29.8)	19,920 (28.7)	22,285 (29.2)	24,281 (29.9)	24,767 (30.2)	27,548 (30.6)	28,297 (30.1)	↗
Dementia	21,630 (4.4)	3090 (4.4)	3466 (4.5)	3631 (4.5)	3541 (4.3)	3911 (4.3)	3991 (4.2)	=
Chronic pulmonary disease	80,759 (16.4)	10,835 (15.6)	12,109 (15.9)	13,237 (16.3)	13,073 (16)	15,206 (16.9)	16,299 (17.3)	↗
Diabetes without chronic complication	81,220 (16.5)	11,171 (16.1)	11,832 (15.5)	13,319 (16.4)	13,739 (16.8)	15,467 (17.2)	15,692 (16.7)	↗
Diabetes with chronic complications	46,219 (9.4)	6735 (9.7)	7175 (9.4)	7762 (9.6)	7490 (9.1)	8519 (9.5)	8538 (9.1)	↘
Renal disease	61,383 (12.5)	7610 (11)	8875 (11.6)	10,003 (12.3)	10,266 (12.5)	12,231 (13.6)	12,398 (13.2)	↗
Any malignancy	111,501 (22.6)	15,377 (22.1)	17,038 (22.3)	18,309 (22.6)	18,709 (22.8)	20,778 (23.1)	21,290 (22.6)	↗
Moderate or severe liver disease	36,692 (7.4)	5306 (7.6)	5706 (7.5)	6115 (7.5)	6187 (7.6)	6559 (7.3)	6819 (7.2)	↘
Metastatic solid tumor	37,339 (7.6)	4734 (6.8)	5516 (7.2)	6033 (7.4)	6311 (7.7)	7271 (8.1)	7474 (7.9)	↗
Charlson score, median (IQR)	2 [1; 4]	2 [1; 4]	2 [1; 4]	2 [1; 4]	2 [1; 4]	2 [1; 4]	2 [1; 4]	↗
Illness severity								
Neurological organ failure	132,587 (26.9)	16,159 (23.3)	19,360 (25.4)	21,817 (26.9)	22,332 (27.3)	25,054 (27.9)	27,865 (29.6)	↗
Renal organ failure	214,785 (43.6)	29,032 (41.8)	32,334 (42.3)	34,910 (43)	36,481 (44.6)	39,981 (44.5)	42,047 (44.7)	↗
Respiratory organ failure	227,706 (46.2)	32,439 (46.7)	35,664 (46.7)	37,638 (46.4)	38,094 (46.5)	40,982 (45.6)	42,889 (45.5)	↘
Number of organ failure, median (IQR)	4 [3; 5]	4 [3; 5]	4 [3; 5]	4 [3; 5]	4 [3; 5]	4 [3; 5]	4 [3; 5]	=
SAPS II score, median (IQR) (miss = 83,156)	51 [38; 67]	51 [38; 67]	51 [38; 67]	51 [38; 67]	51 [38; 67]	51 [38; 67]	51 [38; 68]	↗
Outcomes								
Hospital LOS (days) (median [IQR])	20 [8; 39]	20 [8; 40]	20 [8; 39]	20 [8; 39]	20 [8; 39]	19 [8; 38]	19 [8; 37]	↘
ICU LOS (days) (median [IQR]) (missing = 133,828)	8 [3; 18]	9 [3; 20]	9 [3; 19]	8 [3; 19]	8 [3; 18]	8 [3; 18]	8 [3; 17]	↘
Discharge to home	199,613 (40.5)	27,067 (39)	30,521 (40)	32,755 (40.4)	32,810 (40.1)	37,433 (41.6)	39,027 (41.4)	↗
Hospital mortality	222,709 (45.2)	32,091 (46.2)	34,967 (45.8)	37,007 (45.6)	37,146 (45.4)	39,872 (44.3)	41,626 (44.2)	↘
Cost (€), median (IQR)	16,449 [7336; 29360]	17,261.7 [7604; 30765]	16,129.2 [6918; 29647]	15,886 [6889; 29107]	16,538.6 [7387; 29489]	16,640.1 [7560; 29172]	16,364.6 [7509; 28504]	↘

^a^Test of trend or linear regression for values from 2010 to 2015; ↗ a significant increase is observed with a *p* value < 0.05; ↘ a significant decrease is observed with a *p* value < 0.05; ‘ = ’ no significant trend is observed; *SAPS II* Simplified Acute Physiology Score II; *LOS* length of stay, *ICU* intensive care unit

#### LOS and costs of care

The median hospital and ICU LOS of patients with sepsis were, respectively, 17 days (IQR: 7; 34) and 8 days (IQR: 3; 17) and the cost of the median hospital stay cost amounted to €11,400 (IQR: 5036; 24,364). Hospital and ICU LOS decreased significantly over time, but not the cost of care (Table 1). Septic patients not admitted to the ICU were different from those admitted: they were older (77 years [64; 85]), had more dementia (9%), were more often admitted to the emergency department (32%), were less severely ill and had a shorter hospital LOS and lower cost of care (Additional file 1: Tables S6, S7).

The median hospital and ICU LOS of septic shock patients was, respectively, 20 days (IQR: 8; 39) and 8 days (IQR: 3; 18) and the median hospital stay costs amounted to €16,439 (IQR: 7339; 29,360). The hospital and ICU LOS decreased significantly over time, as did the associated cost of care (Table 2). Similar results were found in the subgroups of patients admitted to the ICU and those included with explicit definitions. Patients with septicemia tended to be more severely ill, with a longer ICU and hospital LOS that increased significantly over time. Finally, septic shock patients not admitted to the ICU were different from those admitted to ICU: they were older (77 years [64; 85]), had more cancer (24%) and dementia (10%), were more often admitted to the emergency department (40%), and had a shorter hospital LOS and lower cost of care, possibly owing to pre-mortem misdiagnosis or early limitation of care (Additional file 1: Tables S8–S10).

#### Hospital mortality and hospital case fatalities

The standardized mortality rate in France for sepsis implicitly defined increased significantly from 70 to 86 deaths per 100,000 inhabitants from 2010 to 2015, and from 62 to 75 deaths per 100,000 inhabitants for patients with septic shock implicitly defined. Similar trends were observed regardless of the sepsis or septic shock definitions used. However, no temporal trends were observed for patients with septicemia. CFR remained quite stable around 34% over time for sepsis with an implicit definition, but decreased from 50 to 48% from 2010 to 2015 for sepsis with an explicit definition and for patients with septicemia. CFR increased significantly from 24 to 32% for septic patients not admitted to the ICU. CFR for septic shock using an implicit definition decreased significantly from 46 to 44% from 2010 to 2015, but remained quite stable, around 51%, when an explicit definition was used. The CFR of septic shock patients with septicemia decreased significantly from 47 to 42% while that of septic shock patients not admitted to the ICU increased significantly, from 59 to 64% (Figs. 2, 3, Tables 1, 2, Additional file 1: Tables S2, S3, S5–S7). The adjusted risk of death decreased significantly independently over time by − 0.33% per year (IQR: − 0.40; − 0.27) for patients with septic shock, but not for those with sepsis (0.08% [− 0.04; 0.20] of deaths per year) (Figs. 2, 3, Additional file 1: Table S11).

## Discussion

This in-depth evaluation of sepsis and septic shock in France from 2010 to 2015 using information from the French national hospital administrative database that records all hospital stays showed an increased incidence of sepsis and septic shock and a decrease in the associated risk of death limited to patients with septic shock. These results should be interpreted in light of our subgroup analyses and the current literature and deserve a few comments.

First, we found an increase in the incidence of sepsis and septic shock in the whole cohort and most of our subgroups. Several studies focusing on sepsis (Additional file 1: Table S1) [7, 9, 12–21] also reported an increased incidence. For instance, *Fleischmann-Struzek *et al. [7] reported an incidence of sepsis that increased from 108 per 100,000 population in 2010 to 158 in 2015. As neither the identification codes nor the reimbursement strategies were modified from 2010 to 2015 in France, the increased incidence of sepsis or septic shock could have been related to an actual increase in the disease incidence due to greater risk factors for sepsis such as the ageing of the population, the increasing burden of comorbidities or the increasing rate of resistances to antibiotics. However, we believe that much of the increase could also be secondary to better identification and coding [22]. It is unfortunately impossible to directly determine how many patients were captured due to better identification. The solution might be to work on detailed clinical data extracted from the electronic health record systems of hospitals [22]. However, these data are not yet available for all hospital stays in France. Nevertheless, it is important to underline that the incidence of sepsis or septic shock due to septicemia has remained fairly stable over time, which indirectly supports a global increase in recognition of other sepsis. The recognition of other sepsis than septicemia could be related to the improvement in the diagnosis of sepsis thanks to successive surviving sepsis campaigns [3, 23], to an improved identification of the pathogens involved or to the rise in electronic medical records and hospital coding systems, which has led to the inclusion of more patients with perhaps less severe septic shock [18, 24, 25].

Septic shock, with a death rate of 45%, is associated with a high risk of mortality. We observed a decrease in the adjusted risk of death by − 0.33% (IQR: − 0.4; − 0.27) per year. This decrease was also found in patients with most severe form of sepsis, i.e., those admitted to the ICU or with bacteremia. Those varying results prompt several remarks. First, to date most studies have reported a decrease in the risk of death over time, from 0.6% per year to 17% per year [7, 9, 12–21]. For instance, *Kadri *et al. [24] reported similar results for septic shock patients, with a decrease in the death rate of around 1.22% per year, from 48.3% in 2005 to 39.3% in 2014. Similar results were also observed among patients with sepsis [26]. Our results, however, were the first to be obtained after adjustment, and compared to those from other countries the decrease although quite smaller was probably more reflective of reality. Our findings could be explained by an improvement in care over time thanks to earlier detection and identification of pathogens, and earlier specific treatment included in care bundles [25, 27]. In contrast, mortality increased in patients not admitted to the ICU regardless of their disease severity. Such results could be explained by pre-mortem misdiagnosis, suboptimal care or early limitation of care. The implicit definition of sepsis using ICD-10 codes included heterogeneous septic patients, almost half of whom were not admitted to the ICU and this could explain why the risk of death was unchanged when all septic patients were considered together.

Finally, we calculated an average cost per hospital stay for septic shock in France of €16,449 compared to the sum of €17,261 in 2010 and the current cost of €16,365. This decrease in costs could have been due to a shorter hospital LOS, mainly because of a shorter ICU LOS, which in turn could be related to the inclusion of older patients with more comorbidities and earlier limitation of care or to earlier discharge of patients to rehabilitation. Another contributing factor could have been an overall improvement in care, with reduction in the time spent on organ support such as ventilation and subsequent reduction in sedation. It is unfortunately difficult to interpret this reduction because of all the confounding factors to be taken into account.

Our work has several strengths. Use of the PMSI allowed all patients with sepsis and septic shock admitted to French hospitals to be included in the study, which with one of the highest number of sepsis and septic shock cases ever recorded gives an accurate picture of the burden they represent for a country with a high standard of care, high hospital bed availability, an integrated healthcare organization structure, and similar admission policies for all patients thanks to its public insurance system. Analysis of a period of time during which diagnostic codes and insurance policies were unchanged ensured that the rise of coding was kept to a minimum. In addition, all procedure codes, diagnostic codes, and the SAPS II scores in the subgroup of ICU patients were taken into account to provide a comprehensive description of septic shock that includes adjustment for mortality risk to a degree not previously attained. We also analyzed hospital stays from admission to death or discharge, taking into account all potential transfers, and adjusting for readmissions, which has not been performed before in similar studies.

Our study has several limitations, mostly related to the nature of the database: the specificity of the coding, the rise in coding due to better recognition and coding, missing data due to billing reasons, and the lack of detailed clinical, paraclinical and drug exposure data. Thus, it was not possible to compare our inclusion criteria with those based on clinical data using the sepsis 3.0 definitions as done elsewhere [28, 29] to determine whether our increase was related to coding and to assess the number of misdiagnosed patients. Nor was it possible to differentiate primary infections from nosocomial infections, to know whether limitations of care were achieved or to take into account discharges to a hospice. Finally, our study did not allow causal inferences between sepsis and/or septic shock and death.

## Conclusions

In conclusion, we showed that sepsis is a common and frequently fatal condition and is associated with high healthcare expenses. In line with other studies based on administrative data, we observed an increased trend in the incidence of sepsis probably due in large part to a better recognition and coding of the infection. We confirmed that improvement in the risk of death for septic shock patients is around 0.3% per year when case-mix and severity scores are taken into account. Similar results were observed for septic patients admitted to the ICU and those with septicemia, suggesting the benefit of being taken into intensive care. Research on sepsis should be actively undertaken in order to sustain the trend in improved outcomes. Lastly, the decline in mortality rates of septic shock is unclear and warrants further studies.

## Supplementary information

**Additional file 1.** Additional figures and tables.

## Data Availability

The datasets used and/or analyzed during the current study are available from the corresponding author upon reasonable request.

## Abbreviations

ICU: Intensive care unit
ICD: International Classification of Diseases;
PMSI: Programme de Médicalisation des Systèmes d'Information
SAPS: Simplified Acute Physiology Score
CCAM: Classification Commune des Actes Médicaux
LOS: Length of stay
GHM: Groupe Homogène de Malades
CFR: Case-fatality rates
IQR: Interquartile range
