# Supplementary material for: Sepsis and septic shock in France: incidences, outcomes and costs of care
Source: Ann Intensive Care. 2020 Oct 20;10:145. doi: 10.1186/s13613-020-00760-x (PMC7575668; doi:10.1186/s13613-020-00760-x)
Supplement: Supplementary file 1 — Additional file 1. Additional figures and tables. [file 13613_2020_760_MOESM1_ESM.doc]

Table S1: Summary of the recent Register-base studies, using ICD coding strategies, dealing with incidence, outcome and associated costs of care of patients with sepsis or septic shock.

| Auteur | Period | Country/Database | Inclusion criteria | Number of patients | Incidence | | Mortality | | Case fatality | | Cost ($) |
| --- | --- | --- | --- | --- | --- | --- | --- | --- | --- | --- | --- |
|  |  |  |  |  | Per 100,000 pop | Trends(/y) | Per 100,000 pop | Trends(/y) | % | Trends(/y) |  |
| Angus [1] | 1995 | US/Multicentric | - Implicit : Inf A + MOF | 192,980 | 300 | ↑ 1.5% |  |  | 28.6 |  | 22,100 |
| Martin [2] | 1979 - 2000 | US/NIS | - Implicit : Inf B | 10,319,418 | from 82.7 to 240.4 | ↑ 8.7% |  |  | from 27.8 to 17.9 |  |  |
| Wang [3] | 2001–2004 | US/NCHS | - Implicit : Inf A + MOF | 2,282,000 |  |  |  |  |  |  |  |
| Dombrovskiy [4] | 1993 to 2003 | US/NIS | - Implicit : Inf C + MOF | 2,857,476 | from 66.8 to 132 | ↑ 8.2% | from 30.3 to 49.7 | ↑5.6% | from 45.0 to 37.7 |  |  |
| Yang [5] | 2004-2007 | Singapor/GHAB | - Implicit : Inf C | 6,926 |  |  |  |  |  |  |  |
| Kumar[6] | 2000-2007 | US/NIS | - Implicit : Inf C + MOF | from 300,270 to 781,725 | from 143 to 343 | ↑ 16.5% |  |  | from 39.6 to 27.3 |  |  |
| Lagu [7] | 2007 | US/NIS | - Implicit: Inf C or A  + MOF | C+ MOF: 719,099  A + MOF: 1,825,758  A or C + MOF: 2.5 million | C+MOF: 303  A+MOF: 771  C or A+MOF: 1074 |  |  |  | C+MOF: 29  A+MOF: 8  A or C+MOF: 14 |  | C+MOF: 19,390  A+MOF: 11,521  A or C+MOF: 13,342 |
| Lagu [8] | 2003-2007 | US/NIS | - Implicit : Inf C + MOF | 2,899,917 | from 200 to 300 |  | from 75 to 87 | ↑ 16% | from 37 to 29 |  | from 20,210 to 19,330 |
| Bouza [9] | 2006-2011 | Spain/MBDS | - Implicit: Inf C + MOF  - Explicit: Sepsis | 240,939 | 86.97  from 63.9 to 105.5 | ↑ 8.6% | 37.1  from 32.1 to 45.3 | ↑ 6% | from 49.3 to 41.9 | ↓ 3.5% |  |
| Ani [10] | 1999-2008 | US/NIS | - Implicit : Inf C + MOF |  |  |  |  |  | 32.9  from 40.0 to 27.8 |  |  |
| Stoller [11] | 2008-2012 | US/NIS | - Implicit: Inf C + MOF  - Explicit: Sepsis | 6,067,789 | from 346 to 436 |  |  |  | from 22.2 to 17.3 |  | 2008: 55,544  2011: 57,987  2012: 55,749 |
| Gohil [12] | 2000-2010 | US/California Mandatory  Hospital Discharge Dataset | - Implicit: Inf C + MOF  - Explicit: Septicemia,  sepsis, sepsis,  septic shock. | 635,780 | from 7,940 and 7,912 |  |  |  |  |  |  |
| Kadri [13] | 2005-2014 | US/University health system consortium | -Explicit: Sepsis, Septic shock | 82,350 |  |  |  |  | Sepsis:  from 48.3 to 39.3  Septic shock:  from 54.9 to 50.7 | Sepsis:  ↓ 1.22%  Septic shock:  ↓ 0.60% |  |
| Rhee[14] | 2003-2009 | US/Massachusetts General Hospital and Brigham and Women’s Hospital | - Implicit: Inf A + MOF  - Explicit: Sepsis,  septic shock. | A + MOF: 69,075  Explicit : 11,096 |  |  |  |  | A + MOF:  from 17.6 to 12.6  Explicit:  from 50.0 to 32.2 | A + MOF: ↓ 5.4%  Explicit: ↓17.3% |  |
| Elfeky [15] | 2009-2012 | US/NIS | - Implicit : Inf B | 1,303,640  from 299,992 to 333,965 |  | ↑ 11% |  |  |  |  | from 45,605 to 44,164 |
| Yébenes [16] | 2008-2012 | Spain/CatSalut | - Implicit : Inf A + MOF | 82,000 | 212.7  from 167.2 to 261.8 |  |  |  | from 23.7 to 19.7 |  |  |
| Rhee [17] | 2009-2014 | US/multicentric | - Implicit: Inf A + MOF  - Explicit: Sepsis,  septic shock | Sepsis : 173,690  Septic shock : 27,502 |  | ↑ 0.6% |  |  | 15.0 | ↓ 3.3% |  |
| Lee [18] | 2002 - 2012 | Tawain/health insurance claims data | - Implicit : Inf A + MOF | 1,259,578 | 637.8 to 772.1 | ↑ 21.1% |  |  | Sepsis: 23.2,  from 23.3 to 17.9;  Septic shock: 38.0,  from 40.5 to 33.2 |  |  |
| Fleischmann-Struzek[19] | 2010-2015 | Germany | -Implicit : Wider Inf A + MOF  -Explicit sepsis | Exp: 136 542  Imp: 1 166 061 | Exp: from 108 to 158  Imp: from 942 to 1336 | Exp:↑7.9%  Imp:↑7.3% | Exp: 51 to 65 |  | Exp: from 47.8 to 41.7% |  |  |

Abbreviation: Inf: infection; MOF: Multiple organ failure; NIS: Nationwide Inpatient Sample; NCHS: National Center for Health Statistics; GHAB: General Hospital Administrative Database; MBDS: Minimum Basic Data Set; CatSalut: Catalan Health System; Infection A, B or C: see below. Codes for implicit or explicit definitions: see below.

**Codes used for the “Implicit” definitions**

**A: A Bacterial or Fungal Infection (“infection or septicemia codes”):**

001, Cholera; 002, Typhoid/paratyphoid; fever; 003, Other salmonella infection; 004, Shigellosis; 005, Other food poisoning; 008, Intestinal infection not otherwise classified; 009, Ill-defined intestinal infection; 010, Primary tuberculosis infection; 011, Pulmonary tuberculosis; 012, Other respiratory tuberculosis; 013, Central nervous system tuberculosis; 014, Intestinal tuberculosis; 015, Tuberculosis of bone and joint; 016, Genitourinary tuberculosis; 017, Tuberculosis not otherwise classified; 018, Miliary tuberculosis; 020, Plague; 021, Tularemia; 022, Anthrax; 023, Brucellosis; 024, Glanders; 025, Melioidosis; 026, Rat-bite fever; 027, Other bacterial zoonoses; 030, Leprosy; 031, Other mycobacterial disease; 032, Diphtheria; 033, Whooping cough; 034, Streptococcal throat/scarlet fever; 035, Erysipelas; 036, Meningococcal infection; 037, Tetanus; 038, Septicemia; 039, Actinomycotic infections; 040, Other bacterial diseases; 041, Bacterial infection in other diseases not otherwise specified; 090, Congenital syphilis; 091, Early symptomatic syphilis; 092, Early syphilis latent; 093, Cardiovascular syphilis; 094, Neurosyphilis; 095, Other late symptomatic syphilis; 096, Late syphilis latent; 097, Other and unspecified syphilis; 098, Gonococcal infections; 100, Leptospirosis; 101, Vincent’s angina; 102, Yaws; 103, Pinta; 104, Other spirochetal infection; 110, Dermatophytosis; 111, Dermatomycosis not otherwise classified or specified; 112, Candidiasis; 114, Coccidioidomycosis; 115, Histoplasmosis; 116, Blastomycotic infection; 117, Other mycoses; 118, Opportunistic mycoses; 320, Bacterial meningitis; 322, Meningitis, unspecified; 324, Central nervous system abscess; 325, Phlebitis of intracranial sinus; 420, Acute pericarditis; 421, Acute or subacute endocarditis; 451, Thrombophlebitis; 461, Acute sinusitis; 462, Acute pharyngitis; 463, Acute tonsillitis; 464, Acute laryngitis/tracheitis; 465, Acute upper respiratory infection of multiple sites/not otherwise specified; 481, Pneumococcal pneumonia; 482, Other bacterial pneumonia; 485, Bronchopneumonia with organism not otherwise specified; 486, Pneumonia, organism not otherwise specified; 491.21, Acute exacerbation of obstructive chronic bronchitis; 494, Bronchiectasis; 510, Empyema; 513, Lung/mediastinum abscess; 540, Acute appendicitis; 541, Appendicitis not otherwise specified; 542, Other appendicitis; 562.01, Diverticulitis of small intestine without hemorrhage; 562.03, Diverticulitis of small intestine with hemorrhage; 562.11, Diverticulitis of colon without hemorrhage; 562.13, Diverticulitis of colon with hemorrhage; 566, Anal and rectal abscess; 567, Peritonitis; 569.5, Intestinal abscess; 569.83, Perforation of intestine; 572.0, Abscess of liver; 572.1, Portal pyemia; 575.0, Acute cholecystitis; 590, Kidney infection; 597, Urethritis/urethral syndrome; 599.0, Urinary tract infection not otherwise specified; 601, Prostatic inflammation; 614, Female pelvic inflammation disease; 615, Uterine inflammatory disease; 616, Other female genital inflammation; 681, Cellulitis, finger/toe; 682, Other cellulitis or abscess; 683, Acute lymphadenitis; 686, Other local skin infection; 711.0, Pyogenic arthritis; 730, Osteomyelitis; 790.7, Bacteremia; 996.6, Infection or inflammation of device/graft; 998.5, Postoperative infection; 999.3, Infectious complication of medical care not otherwise classified

**B: Septicemia and fungemia 1**

038, septicemia; 020.0, septicemic; 790.7, bacteremia; 117.9, disseminated fungal infection; 112.5, disseminated candida infection; 112.81, disseminated fungal endocarditis

**C: Septicemia and fungemia 2**

038.0, streptococcal septicemia; 038.1, staphylococcal septicemia; 038.2, pneumococcal septicemia; 038.3, septicemia due to anaerobies; 038.4, septicemia due to other Gram-negative organisms; 038.8, other specified septicemias; 038.9, unspecified septicemia; 003.1, salmonella septicemia; 020.2, septicemic plague; 022.3, anthrax septicemia; 036.2, meningococcal septicemia; 036.3, Waterhouse-Friderichsen syndrome; 054.5, herpetic septicemia; 098.89, gonococcemia; 112.5, systemic candidiasis; 995.91, systemic inflammatory response syndrome due to infectious process without organ, dysfunction; 995.92, systemic inflammatory response syndrome due to infectious process with organ dysfunction; 785.52, septic shock

**Codes used for the “Explicit” definitions**

septicemia (038x), sepsis (995.91), sepsis (995.92), septic shock (785.52)

Table S2: International Classification of diseases, 10th revision (ICD-10), codes used for the identifications of infections, organ failures and comorbidities

| Label | ICD-10 code |
| --- | --- |
| Infection | A000-B99, G000, G00, G001, G002, G003, G008, G009, G01, G02, G020, G021, G028, G04, G042, G049, G050, G051, G052, G06, G060, G061, G062, G08, H030, H031, H054, H061, H130, H131, H132, H133, H138, H600, H601, H602, H603, H620, H621, H622, H623, H670, H671, H68, H680, H750, H758, I010, I011, I012, I090, I091, I092, I30, I301, I309, I320, I321, I33, I330, I339, I38, I39, I398, I40, I400, I401, I408, I409, I41, I410, I411, I412, I418, I430, I514, I681, I891, I980, I981, J00, J01X, J02X, J03X, J04X, J05X, J06X, J09, J10X, J11X, J12X, J13, J14, J15X, J16X, J17X, J180, J181, J182, J188, J189x, J18X, J20, J200, J201, J202, J203, J204, J205, J206, J207, J208, J209, J21, J210, J211, J218, J219, J22, J36, J390, J391, J40, J440, J85, J850, J851, J852, J853, J86, J860, J869, J953, J961, J982, J983, K046, K047, K050, K052, K103, K113, K122, K230, K231, K238, K2380, K2381, K35, K350, K351, K352, K353, K358, K359, K36, K37, K431, K434, K437, K441, K451, K461, K550, K551, K558, K559, K570, K572, K574, K578, K61, K61, K610, K610, K611, K611, K612, K612, K613, K613, K614, K614, K630, K631, K65, K650, K658, K659, K66, K67, K670, K671, K672, K673, K678, K750, K751, K764, K770, K800, K801, K803, K804, K81, K810, K818, K819, K821, K822, K823, K830, K831, K832, K833, K8700, K930, K93820, K93840, L01, L010, L011, L02, L020, L021, L022, L023, L024, L028, L029, L03, L030, L031, L032, L033, L038, L039, L04, L040, L041, L042, L043, L048, L049, L05, L050, L059, L08, L080, L081, L088, L089, L303, M00, M000, M0000, M0001, M0002, M0003, M0004, M0005, M0006, M0007, M0008, M0009, M001, M0010, M0011, M0012, M0013, M0014, M0015, M0016, M0017, M0018, M0019, M002, M0020, M0021, M0022, M0023, M0024, M0025, M0026, M0027, M0028, M0029, M008, M0080, M0081, M0082, M0083, M0084, M0085, M0086, M0087, M0088, M0089, M009, M0090, M0091, M0092, M0093, M0094, M0095, M0096, M0097, M0098, M0099, M01, M010, M0100, M0101, M0102, M0103, M0104, M0105, M0106, M0107, M0108, M0109, M011, M0110, M0111, M0112, M0113, M0114, M0115, M0116, M0117, M0118, M0119, M012, M0120, M0121, M0122, M0123, M0124, M0125, M0126, M0127, M0128, M0129, M013, M0130, M0131, M0132, M0133, M0134, M0135, M0136, M0137, M0138, M0139, M014, M0140, M0141, M0142, M0143, M0144, M0145, M0146, M0147, M0148, M0149, M015, M0150, M0151, M0152, M0153, M0154, M0155, M0156, M0157, M0158, M0159, M016, M0160, M0161, M0162, M0163, M0164, M0165, M0166, M0167, M0168, M0169, M018, M0180, M0181, M0182, M0183, M0184, M0185, M0186, M0187, M0188, M0189, M462, M4620, M4621, M4622, M4623, M4624, M4625, M4626, M4627, M4628, M4629, M463, M4630, M4632, M4633, M4634, M4635, M4636, M4637, M4638, M4639, M464, M4640, M4642, M4643, M4644, M4645, M4646, M4647, M4648, M4649, M465, M4650, M4651, M4652, M4653, M4654, M4655, M4656, M4657, M4658, M4659, M490, M4900, M4901, M4902, M4903, M4904, M4905, M4906, M4907, M4908, M4909, M491, M4910, M4911, M4912, M4913, M4914, M4915, M4916, M4917, M4918, M4919, M492, M4920, M4921, M4922, M4923, M4924, M4925, M4926, M4927, M4928, M4929, M493, M4930, M4931, M4932, M4933, M4934, M4935, M4936, M4937, M4938, M4939, M60, M600, M6000, M6001, M6002, M6003, M6004, M6005, M6006, M6007, M6008, M6009, M630, M631, M632, M65, M650, M6500, M6501, M6502, M6503, M6504, M6505, M6506, M6507, M6508, M6509, M651, M6510, M6511, M6512, M6513, M6514, M6515, M6516, M6517, M6518, M6519, M711, M7110, M7111, M7112, M7113, M7114, M7115, M7116, M7117, M7118, M7119, M726, M7260, M7261, M7262, M7263, M7264, M7265, M7266, M7267, M7268, M7269, M730, M7300, M7301, M7302, M7303, M7304, M7305, M7306, M7307, M7308, M7309, M731, M7310, M7311, M7312, M7313, M7314, M7315, M7316, M7317, M7318, M7319, M86, M860, M8600, M8601, M8602, M8603, M8604, M8605, M8606, M8607, M8608, M8609, M861, M8610, M8611, M8612, M8613, M8614, M8615, M8616, M8617, M8618, M8619, M862, M8620, M8621, M8622, M8623, M8624, M8625, M8626, M8627, M8628, M8629, M864, M8640, M8641, M8642, M8643, M8644, M8645, M8646, M8647, M8648, M8649, M865, M8650, M8651, M8652, M8653, M8654, M8655, M8656, M8657, M8658, M8659, M868, M8680, M8681, M8682, M8683, M8684, M8685, M8686, M8687, M8688, M8689, M869, M8690, M8691, M8692, M8693, M8694, M8695, M8696, M8697, M8698, M8699, M90, M900, M9000, M9001, M9002, M9003, M9004, M9005, M9006, M9007, M9008, M9009, M901, M9010, M9011, M9012, M9013, M9014, M9015, M9016, M9017, M9018, M9019, M902, M9020, M9021, M9022, M9023, M9024, M9025, M9026, M9027, M9028, M9029, N10, N110, N111, N12, N136, N151, N160, N290, N291, N30, N300, N309, N330, N34, N340, N342, N369, N390, N410, N411, N412, N413, N419, N431, N45, N450, N459, N70, N700, N709, N710, N719, N72, N73, N730, N731, N732, N733, N734, N735, N739, N740, N741, N742, N743, N744, N751, N760, N761, N762, N763, N764, N770, N771, O040, O045, O050, O080, O23, O230, O231, O232, O233, O234, O235, O239, O411, O740, O752, O753, O85, O86, O860, O861, O862, O863, O864, O868, O883, O91, O910, O911, O98, O980, O981, O982, O983, O984, O985, O986, O987, O988, O989, R02, R50, R500, R501, R508, R509, R55, R568, R57, R572, R578, R579, R619, R65, R650, R651, R659, R680, R75, R827, R835, R845, R855, R865, R875, T793, T802, T814, T826, T827, T835, T836, T845, T846, T847, T857, T874, T880, U04, U049 |
| Type of pathogens | |
| Fungal infection | B35, B350, B351, B352, B353, B354, B355, B356, B358, B359, B36, B360, B361, B362, B363, B368, B369, B37, B370, B371, B372, B373, B373+, B374, B375, B375+, B376, B376+, B377, B378, B379, B38, B380, B381, B382, B383, B384, B384+, B387, B388, B389, B39, B390, B391, B392, B393, B394, B395, B399, B40, B400, B401, B402, B403, B407, B408, B409, B41, B410, B417, B418, B419, B42, B420, B420+, B421, B427, B428, B429, B43, B430, B431, B432, B438, B439, B44, B440, B441, B442, B447, B448, B449, B45, B450, B451, B452, B453, B457, B458, B459, B46, B460, B461, B462, B463, B464, B465, B468, B469, B47, B470, B471, B479, B48, B480, B481, B482, B483, B484, B487, B488, B49, C840, J67, L22, P375, B35 |
| Virus infection | A080, A081, A082, A083, A084, A60, A600, A601, A609, A630, A80, A800, A801, A802, A803, A804, A809, A81, A810, A811, A812, A818, A819, A82, A820, A821, A829, A83, A830, A831, A832, A833, A834, A835, A836, A838, A839, A84, A840, A841, A848, A849, A85, A850, A850+, A851, A851+, A852, A858, A86, A87, A870, A870+, A871, A871+, A872, A878, A879, A88, A880, A881, A888, A89, A90, A91, A92, A920, A921, A922, A923, A924, A928, A929, A93, A930, A931, A932, A938, A94, A95, A950, A951, A959, A96, A960, A961, A962, A968, A969, A980, A981, A982, A983, A984, A985, A988, A99, B000, B001, B002, B003, B004, B005, B007, B008, B009, B010, B011, B012, B018, B019, B020, B021, B022, B023, B027, B028, B029, B03, B04, B050, B051, B052, B053, B054, B058, B059, B060, B068, B069, B07, B080, B081, B082, B083, B084, B085, B088, B09, B150, B159, B160, B161, B162, B169, B17.9, B170, B171, B172, B178, B180, B181, B182, B188, B189, B190, B199, B200, B201, B202, B203, B204, B205, B206, B207, B208, B209, B210, B211, B212, B213, B217, B218, B219, B220, B221, B222, B227, B230, B231, B232, B238, B24, B24+0, B24+1, B24+9, B250, B251, B252, B258, B259, B260, B261, B262, B263, B268, B269, B270, B271, B278, B279, B300, B301, B302, B303, B308, B309, B330, B331, B332, B333, B338, B340, B341, B342, B343, B344, B348, B349, B91, B941, B942, B970, B971, B972, B973, B974, B975, B976, B977, B978, G020, G051, I411, J09, J10, J100, J101, J108, J11, J110, J111, J118, J12, J120, J121, J122, J123, J128, J129, J171, J203, J204, J205, J206, J207, J210, J211, K2380, K8700, K93820, K93840, M014, M0140, M0141, M0142, M0143, M0144, M0145, M0146, M0147, M0148, M0149, M015, M0150, M0151, M0152, M0153, M0154, M0155, M0156, M0157, M0158, M0159, O984, O985, O987 |
| Gram negative bacilli | A00, A000, A001, A009, A01, A010, A011, A012, A013, A014, A02, A020, A021, A022, A022+, A028, A029, A03, A030, A031, A032, A033, A038, A039, A04, A040, A041, A042, A043, A044, A045, A046, A053, A20, A200, A201, A202, A203, A207, A208, A209, A21, A210, A211, A212, A213, A217, A218, A219, A23, A230, A231, A232, A233, A238, A239, A24, A240, A241, A242, A243, A244, A25, A250, A251, A259, A26, A260, A267, A268, A269, A280, A281, A282, A37, A370, A371, A378, A379, A413, A415, A430, A438, A44, A440, A441, A448, A449, A480, A481, A482, A484, A492, A57, B961, B962, B963, B964, B965, B966, B9680, B9681, B980, G000, J14, J150, J151, J155, J156, J201, M491, M4910, M4911, M4912, M4913, M4914, M4915, M4916, M4917, M4918, M4919, M492, M4920, M4921, M4922, M4923, M4924, M4925, M4926, M4927, M4928, M4929 |
| Gram positive cocci | A050, A38, A40, A400, A401, A402, A403, A408, A409, A410, A411, A412, A46, A483, A490, A491, A544, A549, B95, B950, B951, B952, B953, B954, B955, B956, B957, B958, G001, G002, G003, J020, J030, J13, J152, J153, J154, J202, L01, L010, L011, M000, M0000, M0001, M0002, M0003, M0004, M0005, M0006, M0007, M0008, M0009, M001, M0010, M0011, M0012, M0013, M0014, M0015, M0016, M0017, M0018, M0019, M002, M0020, M0021, M0022, M0023, M0024, M0025, M0026, M0027, M0028, M0029, M009, M0090, M0091, M0092, M0093, M0094, M0095, M0096, M0097, M0098, M0099, M463, M4630, M4632, M4633, M4634, M4635, M4636, M4637, M4638, M4639, A050 |
| Source of infection | |
| Pulmonary | J09, J10, J100, J101, J108, J11, J110, J111, J118, J12, J120, J121, J122, J123, J128, J129, J13, J14, J15, J150, J151, J152, J153, J154, J155, J156, J157, J158, J159, J16, J160, J168, J17, J170, J171, J172, J173, J178, J18, J180, J181, J182, J188, J189, J20, J200, J201, J202, J203, J204, J205, J206, J207, J208, J209, J21, J210, J211, J218, J219, J22, J40, J440, J85, J850, J851, J852, J853, J86, J860, J869, J953, J961, J982, J983 |
| Intra-abdominal | A090, A099, K047, K050, K052, K103, K113, K122, K230, K231, K238, K2380, K2381, K35, K350, K351, K352, K353, K358, K359, K36, K37, K431, K434, K437, K441, K451, K461, K550, K551, K558, K559, K570, K572, K574, K578, K61, K610, K611, K612, K613, K614, K630, K631, K65, K650, K658, K659, K66, K67, K670, K671, K672, K673, K678, K750, K751, K764, K770, K800, K801, K803, K804, K81, K810, K811, K818, K819, K821, K822, K823, K83, K830, K832, K833, K8700, K930, K93820, K93840, K046, K831 |
| Urinary tract | N10, N080, N110, N111, N12, N136, N151, N160, N290, N291, N30, N300, N301, N302, N303, N304, N308, N309, N33, N330, N338, N34, N340, N341, N342, N343, N369, N37, N370, N378, N390, N41, N410, N411, N412, N413, N418, N419, N431, N45, N450, N459, N512 , N70, N700, N701, N709, N71, N710, N711, N719, N72, N73, N730, N731, N732, N733, N734, N735, N736, N738, N739, N74, N740, N741, N742, N743, N744, N751, N760, N761, N762, N763, N764, N770, N771 |
| Septicemia | A021, A227, A267, A327, A40, A400, A401, A402, A403, A408, A409, A41, A410, A411, A412, A413, A414, A415, A418, A419, A427, A548, T802, T880, B377 |
| Organ failure (OF) | |
| Cardio vascular OF | I95, E86, E872, I951, I958, I959, I981 R55, R571, R572, R578, R579, R65, R651, R659 |
| Haematological OF | D65, D689, D695, D696, D762 |
| Liver OF | R17, K72, K720, K729 |
| Neurological OF | F05, F050, F058, F059, F079, F09, G934, R40, R400, R401, R4018, R402, R4028, R410, R451 |
| Kidney OF | R392, R34, N17, N170, N171, N172, N178, N179, N19 |
| Respiratory OF | J80, J951, J952, J96, J960, J9600, J9601, J9609, J969, J9690, J9691, J9699, R092, R230 |
| Septic shock | R572, R578, R579 |
| Sepsis | R651 |
| **Comorbidities according to an adaptation of the Charlson score**[20] | |
| Myocardial infarction | I20, I200, I201, I208, I209, I21, I210, I211, I212, I213, I214, I219, I22, I220, I221, I228, I229, I23, I230, I231, I232, I233, I234, I235, I236, I238, 'I24, I240, I241, I248, I249, I25, I250, I251, I252, I253, I254, I255, I256, I258, I259 |
| Congestive heart failure | I420, I421, I422, I423, I424, I425, I426, I427, I428, I429, 'I430, I431, I432, I438, I099, I110, I130, I132, I255, P290, I50, I500, I501, I509 |
| Peripheral vascular disease | I700, I701, I702, I708, I709, I7000, I7001, I7020, I7021, I7080, I7081, I7090, I7091, I71, I710, I711, I712, I713, I714, I715, I716, I718, I719, K551, K558, K559, Z958, Z959, I72, I720, I721, I722, I723, I724, I728, I729, I73, I730, I731, I738, I739, I77, I770, I771, I772, I773, I774, I775, I776, I778, I779, I78, I79, I790, I791, I792, I798 |
| Cerebrovascular disease | I60, I600, I601, I602, I603, I604, I605, I606, I607, I608, I609, 'I61, I610, I611, I612, I613, I614, I615, I616, I618, I619, I62, I620, I621, I629, I63, I630, I631, I632, I633, I634, I635, I636, I638, I639, I64, I65, I650, I651, I652, I653, I658, I659, I66, I660, I661, I662, I663, I664, I668, I669, I67, I670, I671, I672, I673, I674, I675, I676, I677, I678, I679, I68, I680, I681, I682, I688, I69, I690, I691, I692, I693, I694, I698, H340, H341, H342, H348, H349, H34, G45, G450, G451, G452, G453, 'G454, G458, G459, G46, G460, G461, G462, G463, G464, G465, G466, G467, G468, I725, I726, I720 |
| Dementia | F001, F00, F000, F001*, F002, F009, F01, F010, F011, F012, F013, F018, F019, F02, F020, F021, F022, F023, F024, F028, F03, F04, F051, G30, G300, G301, 'G301, G308, G309, G310, G311, G312, G318, G319, G32, G320, G328 |
| Chronic pulmonary disease | J40, I278, I279, J40, J41, J410, J411, J418, J42, J43, J430, J431, J432, J438, J439, J44, J440, J441, J448, J449, J450, J451, J458, J459, 'J46, J47, J60, J61, J620, J628, J630, J631, J632, J633, J634, J635, J638, J64, J65, J660, J661, J662, J668, J670, J671, J672, J673, J674, J675, J676, J677, J678, J679, J684, J701, J703, J96, J9610, J96100, J96101, J9611, J96110, J96111, J9619, J96190, J96191, J9690, J9691, J9699 |
| Rheumatic disease | J990, M050, M0500, M0501, M0502, M0503, M0504, M0505, M0506, M0507, M0508, M0509, M051, M0510, M0511, M0512, M0513, M0514, M0515, M0516, M0517, M0518, M0519, M052, M0520, M0521, M0522, M0523, M0524, M0525, M0526, M0527, M0528, M0529, M053, M0530, M0531, M0532, M0533, M0534, M0535, M0536, M0537, M0538, M0539, M058, M0580, M0581, M0582, M0583, M0584, M0585, M0586, M0587, M0588, M0589, M059, M0590, M0591, M0592, M0593, M0594, M0595, M0596, M0597, M0598, M0599, M060, M0600, M0601, M0602, M0603, M0604, M0605, M0606, M0607, M0608, M0609, M061, M0610, M0611, M0612, M0613, M0614, M0615, M0616, M0617, M0618, M0619, M062, M0620, M0621, M0622, M0623, M0624, M0625, M0626, M0627, M0628, M0629, M063, M0630, M0631, M0632, M0633, M0634, M0635, M0636, M0637, M0638, M0639, M064, M0640, M0641, M0642, M0643, M0644, M0645, M0646, M0647, M0648, M0649, M068, M0680, M0681, M0682, M0683, M0684, M0685, M0686, M0687, M0688, M0689, M069, M0690, M0691, M0692, M0693, M0694, M0695, M0696, M0697, M0698, M0699, M30, M300, M301, M302, M303, M308, M31, M310, M311, M312, M313, M314, M315, M316, M317, M318, M319, M32, M320, M321, M321+, M328, M329, M33, M330, M331, M332, M339, M34, M340, M341, M342, M348, M349, M35, M350, M351, M352, M353, M354, M355, M356, M357, M358, M359, M36, M360, M361, M362, M363, M364, M368, M42, M420, M421, M429, M60, M600, M601, M602, M608, M609, M61, M610, M611, M612, M613, M614, M615, M619, M633, M638 |
| Peptic ulcer disease | K250, K251, K252, K253, K254, K255, K256, K257, K259, K260, K261, K262, K263, K264, K265, K266, K267, K269, K270, K271, 'K272, K273, K274, K275, K276, K277, K279, K280, K281, K282, K283, K284, K285, K286, K287, K289, K221, K223, K226, K25, 'K26, K27, K28, K29, K290, K291, K292, K293, K294, K295, K296, K297, K298, K299 |
| Mild liver disease | B18, B180, B181, B182, B188, B189, K70, K700, K701, K702, K703, K704, K709, K71, K710, K711, K712, K713, K714, K715, K716, K717, K718, K719, K72, K720, K721, K729, K73, K730, K731, K732, K738, K739, K75, K750, K751, K752, K753, K754, K758, K759, K76, K760, K761, K762, K763, K764, K765, K766, K767, K768, K769, K77, K770, K778, Z944 |
| Diabetes without chronic complication | E10, E100, E101, E106, E108, E109, E11, E110, E1100, E1108, E111, E1110, E1118, E116, E118, E119, E1190, E1198, E12, E120, E121, E126, E128, E129, E13, E130, E131, E136, E138, E139, E14, E140, E141, E146, E148, E149, O24, O240, O241, O242, O243, O244, O249 |
| Diabetes with chronic complication | E102, E102+, E103, E103+, E104, E104+, E105, E106, E107, E108, E112, E112+, E1120, E1128, E113, E113+, E1130, E1138, E114, E114+, E1140, E1148, E115, E1150, E1158, E116, E1160, E1168, E117, E1170, E1178, E118, E1180, E1188, E122, E122+, E123, E123+, E124, E124+, E125, E126, E127, E128, E132, E132+, E133, E133+, E134, E134+, E135, E136, E137, E138, E142, E142+, E143, E143+, E144, E144+, E145, E146, E147, E148 |
| Hemiplegia or paraplegia | G041, G114, G80, G800, G801, G802, G803, G804, G808, G809, G81, G810, G811, G819, G82, G820, G821, G822, G823, G824, G825, G83, G830, G831, G832, G833, G834, G838, G838+0, G838+8, G839 |
| Renal disease | N030, I120, I129, I130, I131, I132, I139, I150, I151, N00, N01, N02, N03, N031, N0310, N0319, N032, N033, N0330, N0339, N034, N035, N036, N037, N038, N0380, N0389, N039, N04, N040, N0400, N0409, N041, N042, N043, N044, N045, N046, N047, N048, N049, N05, N050, N051, N052, N053, N054, N055, N056, N057, N058, N059, N10, N11, N110, N111, N118, N119, N12, N13, N130, N131, N132, N133, N134, N135, N136, N137, N138, N139, N14, N140, N141, N142, N143, N144, N15, N150, N151, N158, N159, N16, N160, N161, N162, N163, N164, N165, N168, N17, N170, N171, N172, N178, N179, N18, N180, N181, N182, N183, N184, N185, N188, N189 |
| Any malignancy | C000, C00, C000, C001, C002, C003, C004, C005, C006, C008, C009, C01, C02, C020, C021, C022, C023, C024, C028, C029, C03, C030, C031, C039, C04, C040, C041, C048, C049, C05, C050, C051, C052, C058, C059, C06, C060, C061, C062, C068, C069, C07, C08, C080, C081, C088, C089, C09, C090, C091, C098, C099, C10, C100, C101, C102, C103, C104, C108, C109, C11, C110, C111, C112, C113, C118, C119, C12, C13, C130, C131, C132, C138, C139, C14, C140, C142, C148, C15, C150, C151, C152, C153, C154, C155, C158, C159, C16, C160, C161, C162, C163, C164, C165, C166, C168, C169, C17, C170, C171, C172, C173, C178, C179, C18, C180, C181, C182, C183, C184, C185, C186, C187, C188, C189, C19, C20, C21, C210, C211, C212, C218, C22, C220, C221, C222, C223, C224, C227, C229, C23, C24, C240, C241, C248, C249, C25, C250, C251, C252, C253, C254, C257, C258, C259, C26, C260, C261, C268, C269, C30, C300, C301, C31, C310, C311, C312, C313, C318, C319, C32', C320, C321, C322, C323, C328, C329, C33, C340, C341, C342, C343, C348, C349, C37, C380, C381, C382, C383, C384, C388, C390, C398, C399, C400, C401, C402, C403, C408, C409, C410, C411, C412, C413, C414, C418, C419, C430, C431, C432, C433, C434, C435, C436, C437, C438, C439, C440, C441, C442, C443, C444, C445, C446, C447, C448, C449, C450, C451, C452, C457, C459, C460, C461, C462, C463, C467, C4670, C4671, C4672, C4678, C468, C469, C470, C471, C472, C473, C474, C475, C476, C478, C479, C480, C481, C482, C488, C490, C491, C492, C493, C494, C495, C496, C498, C499, C500, C501, C502, C503, C504, C505, C506, C508, C509, C510, C511, C512, C518, C519, C52, C530, C531, C538, C539, C540, C541, C542, C543, C548, C549, C55, C56, C570, C571, C572, C573, C574, C577, C578, C579, C58, C600, C601, C602, C608, C609, C61, C620, C621, C629, C630, C631, C632, C637, C638, C639, C64, C65, C66, C670, C671, C672, C673, C674, C675, C676, C677, C678, C679, C680, C681, C688, C689, C690, C691, C692, C693, C694, C695, C696, C698, C699, C700, C701, C709, C710, C711, C712, C713, C714, C715, C716, C717, C718, C719, C720, C721, C722, C723, C724, C725, C728, C729, C73, C740, C741, C749, C750, C751, C752, C753, C754, C755, C758, C759, C760, C761, C762, C763, C764, C765, C767, C768, C810, C811, C812, C813, C817, C819, C820, C821, C822, C827, C829, C830, C831, C832, C833, C834, C835, C836, C837, C838, C839, C840, C841, C842, C843, C844, C845, C850, C851, C857, C859, C880, C881, C882, C883, C887, C889, C900, C901, C902, C910, C911, C912, C913, C914, C915, C917, C919, C920, C921, C922, C923, C924, C925, C927, C929, C930, C931, C932, C937, C939, C940, C941, C942, C943, C944, C945, C947, C950, C951, C952, C957, C959, C960, C961, C962, C963, C967, C969, C97 |
| Moderate or severe liver disease | B179, I85, I850, I859, I864, I982, I983, K700, K703, K711, K717, K721, K729, K74x, K76x, K704 |
| Metastatic solid tumor | C77x, C78x, C79x, C80x |
| AIDS/HIV | B20x, B21x, B22x, B23x, B24x |

Table S 3: CCAM codes (French acronym for Classification Commune des Actes Médicaux, i.e., General Coding of Medical Acts) including organ failure supplementation used for the identifications of organ failures.

| Cardio vascular organ failure | EQLF003, EQLF002, DKMD001, DKMD002 |
| --- | --- |
| Haematological organ failure | FELF003 |
| Respiratory organ failure | GLLD003, GLLD004, GLLD008, GLLD012, GLLD015, GLLD019 |
| Neurological organ failure | GELD0040, ABQP001, AAQP003 |
| Kidney organ failure | JVJF002, JVJF005 |

Table S4: Characteristics of study hospitals

| Hospital characteristics | Distribution among study hospitals |
| --- | --- |
|  | No. (%) (n=1413) |
| Public | 884 (62.5) |
| Private | 530 (37.5) |
| Regional hospital | 35 (2.5) |
| General hospital | 450 (32.1) |
| Local hospital | 185 (13.2) |
| Others | 743 (52.6) |

Table S5: Subgroup analyses of septic patients with septicemia defined implicitly using ICD-10 or CCAM. Incidence, main characteristics, outcomes, costs and temporal trends from 2010 to 2015.

| ***Sepsis with septicemia***  ***Implicit definition using CCAM or ICD-10*** | ***All*** | ***2010*** | ***2011*** | ***2012*** | ***2013*** | ***2014*** | ***2015*** | *Trends** |
| --- | --- | --- | --- | --- | --- | --- | --- | --- |
| *Cases* | 141669 | 25361 | 23045 | 22516 | 20969 | 22271 | 27507 |  |
| *Incidence per 100.000 (age-standardized)* |  | 49 | 44 | 42 | 39 | 41 | 49 | = |
| *Deaths per 100.000 (age-standardized)* |  | 21 | 18 | 17 | 15 | 15 | 19 | = |
| *Case fatality (age standardized)* |  | 43 | 40 | 39 | 39 | 38 | 38 | ↘ |
| *1st admission (between 2010 and 2015)* | 130876 (92.4) | 24575 (96.9) | 21622 (93.8) | 20708 (92) | 19106 (91.1) | 20138 (90.4) | 24727 (89.9) | ↘ |
| *Number of admissions, median (IQR)* | 1 [1 ; 1] | 1 [1 ; 1] | 1 [1 ; 1] | 1 [1 ; 1] | 1 [1 ; 1] | 1 [1 ; 1] | 1 [1 ; 1] | = |
| *Readmission (during the year following the last admission)* | 9446 (6.7) | 1554 (6.1) | 1423 (6.2) | 1533 (6.8) | 1430 (6.8) | 1557 (7) | 1949 (7.1) | ↗ |
| *Baseline characteristics* |  |  |  |  |  |  |  |  |
| *Age in years, median (IQR)* | 68 [58 ; 78] | 68 [57 ; 78] | 68 [57 ; 78] | 68 [57 ; 78] | 68 [58 ; 78] | 68 [58 ; 78] | 69 [59 ; 79] | ↗ |
| *Female gender* | 51885 (36.6) | 9590 (37.8) | 8545 (37.1) | 8228 (36.5) | 7526 (35.9) | 8033 (36.1) | 9963 (36.2) | ↘ |
| *Charlson score, median (IQR)* | 3 [1 ; 5] | 2 [1 ; 5] | 3 [1 ; 5] | 3 [1 ; 5] | 3 [1 ; 5] | 3 [1 ; 5] | 3 [1 ; 5] | ↗ |
| Severity of the patient illness |  |  |  |  |  |  |  |  |
| *Cardio vascular organ failure* | 112482 (79.4) | 19085 (75.3) | 18097 (78.5) | 17735 (78.8) | 16813 (80.2) | 18047 (81) | 22705 (82.5) | ↗ |
| *Number of organ failure, median (IQR)* | 4 [3 ; 5] | 4 [3 ; 5] | 4 [3 ; 5] | 4 [2 ; 5] | 4 [2 ; 5] | 4 [2 ; 5] | 4 [2 ; 5] | ↘ |
| *SAPS II score, median (IQR)(miss=27406)* | 51 [39 ; 67] | 52 [40 ; 68] | 51 [39 ; 67] | 51 [39 ; 66] | 51 [38 ; 67] | 51 [38 ; 66] | 51 [38 ; 66] | ↘ |
| *ICU admission* | 119080 (84.1) | 21971 (86.6) | 19536 (84.8) | 18806 (83.5) | 17498 (83.4) | 18532 (83.2) | 22737 (82.7) | ↘ |
| Outcome |  |  |  |  |  |  |  |  |
| *Hospital LOS(days), median (IQR)* | 26 [12 ; 49] | 25 [11 ; 48] | 26 [12 ; 49] | 26 [12 ; 50] | 26 [12 ; 50] | 27 [12 ; 51] | 25 [12 ; 47] | = |
| *ICU LOS(days), median (IQR)(miss=43283)* | 10 [4 ; 23] | 10 [4 ; 23] | 10 [4 ; 24] | 10 [4 ; 23] | 10 [4 ; 23] | 10 [4 ; 24] | 9 [4 ; 22] | = |
| *Difference between hospital and ICU stay(days), median (IQR)* | 15 [5 ; 33] | 14 [3 ; 30] | 15 [5 ; 32] | 16 [5 ; 33] | 17 [6 ; 35] | 17 [6 ; 34] | 16 [6 ; 32] | ↗ |
| *Discharge to home* | 66941 (47.3) | 11022 (43.5) | 10637 (46.2) | 10763 (47.8) | 10042 (47.9) | 11018 (49.5) | 13459 (48.9) | ↗ |
| *Hospital mortality* | 57098 (40.3) | 11053 (43.6) | 9471 (41.1) | 9018 (40.1) | 8333 (39.7) | 8615 (38.7) | 10608 (38.6) | ↘ |
| *Cost (€), median (IQR)* | 18929.6  [8778.2 ; 34437.4] | 19494.6  [8980 ; 34325] | 19136.2  [8663.8 ; 34865.6] | 18940.5  [8680.9 ; 34916.8] | 19325.3  [9042.5 ; 35037.7] | 18928  [8942.1 ; 35104.1] | 17930.4  [8592.7 ; 32984.1] | ↘ |

*test of trend or linear regression for values from 2010 to 2015; ↗ a significant increase is observed with a pvalue < 0.05; ↘ a significant decrease is observed with a pvalue < 0.05; ‘=’ no significant trend is observed ; SAPS II: simplified acute physiology score II; LOS: Length of stay; ICU: Intensive care unit

Table S6: Subgroup analyses of septic patients admitted to ICU defined implicitly using ICD-10 or CCAM. Incidence, main characteristics, outcomes, costs and temporal trends from 2010 to 2015.

| ***Sepsis admitted to ICU***  ***Implicit definition using CCAM or ICD-10*** | ***All*** | ***2010*** | ***2011*** | ***2012*** | ***2013*** | ***2014*** | ***2015*** | *Trends** |
| --- | --- | --- | --- | --- | --- | --- | --- | --- |
| *Cases* | 519049 | 74245 | 80747 | 85670 | 86547 | 94873 | 96967 |  |
| *Incidence per 100.000 (age-standardized)* |  | 144 | 155 | 162 | 161 | 174 | 176 | ↗ |
| *Deaths per 100.000 (age-standardized)* |  | 55 | 58 | 60 | 59 | 63 | 64 | ↗ |
| *Case fatality (age standardized)* |  | 38 | 37 | 37 | 37 | 36 | 36 | ↘ |
| *1st admission (between 2010 and 2015)* | 444612 (85.7) | 70561 (95) | 72422 (89.7) | 74044 (86.4) | 72571 (83.9) | 77640 (81.8) | 77374 (79.8) | ↘ |
| *Number of admissions, median (IQR)* | 1 [1 ; 1] | 1 [1 ; 1] | 1 [1 ; 1] | 1 [1 ; 1] | 1 [1 ; 1] | 1 [1 ; 1] | 1 [1 ; 1] | = |
| *Readmission (during the year following the last admission)* | 58854 (11.3) | 7650 (10.3) | 8325 (10.3) | 9371 (10.9) | 9962 (11.5) | 11256 (11.9) | 12290 (12.7) | ↗ |
| *Baseline characteristics* |  |  |  |  |  |  |  |  |
| *Age in years, median (IQR)* | 68 [58 ; 79] | 68 [56 ; 78] | 68 [57 ; 78] | 68 [57 ; 79] | 69 [58 ; 79] | 68 [58 ; 79] | 68 [58 ; 79] | ↗ |
| *Female gender* | 194586 (37.5) | 27941 (37.6) | 30251 (37.5) | 32257 (37.7) | 32387 (37.4) | 35625 (37.6) | 36125 (37.3) | = |
| *Charlson score, median (IQR)* | 2 [1 ; 5] | 2 [1 ; 4] | 2 [1 ; 4] | 2 [1 ; 5] | 2 [1 ; 5] | 3 [1 ; 5] | 2 [1 ; 5] | ↗ |
| Severity of the patient illness |  |  |  |  |  |  |  |  |
| *Cardio vascular organ failure* | 384391 (74.1) | 51333 (69.1) | 58582 (72.6) | 62731 (73.2) | 64309 (74.3) | 72128 (76) | 75308 (77.7) | ↗ |
| *Number of organ failure, median (IQR)* | 4 [3 ; 5] | 4 [3 ; 4] | 4 [3 ; 5] | 4 [3 ; 5] | 4 [3 ; 5] | 4 [3 ; 5] | 4 [3 ; 5] | = |
| *SAPS II score, median (IQR)* | 48 [36 ; 65] | 48 [36 ; 64] | 48 [36 ; 64] | 48 [36 ; 64] | 48 [36 ; 64] | 48 [36 ; 65] | 49 [36 ; 65] | ↗ |
| *ICU admission* | 519049 (100) | 74245 (100) | 80747 (100) | 85670 (100) | 86547 (100) | 94873 (100) | 96967 (100) |  |
| Outcome |  |  |  |  |  |  |  |  |
| *Hospital LOS(days), median (IQR)* | 21 [10 ; 41] | 22 [10 ; 42] | 22 [10 ; 41] | 22 [10 ; 41] | 22 [10 ; 41] | 21 [10 ; 40] | 21 [10 ; 39] | ↘ |
| *ICU LOS(days), median (IQR)(miss=116074)* | 8 [3 ; 17] | 8 [3 ; 19] | 8 [3 ; 18] | 8 [3 ; 18] | 8 [3 ; 17] | 7 [3 ; 17] | 7 [3 ; 16] | ↘ |
| *Difference between hospital and ICU stay, median (IQR)* | 13 [4 ; 27] | 12 [3 ; 27] | 12 [4 ; 27] | 13 [4 ; 27] | 14 [5 ; 28] | 13 [4 ; 27] | 12 [4 ; 26] | = |
| *Discharge to home* | 247595 (47.7) | 34192 (46.1) | 38061 (47.1) | 40910 (47.8) | 41311 (47.7) | 46501 (49) | 46620 (48.1) | ↗ |
| *Hospital mortality* | 193825 (37.3) | 28499 (38.4) | 30507 (37.8) | 32104 (37.5) | 32159 (37.2) | 34647 (36.5) | 35909 (37) | ↘ |
| *Cost (€), median (IQR)* | 17347.7  [8667.8 ; 30019.7] | 18069.8  [8892.2 ; 31351.5] | 17239.4  [8451.6 ; 30345.9] | 16989.5  [8382.1 ; 29786.5] | 17384.4  [8713.5 ; 30119.7] | 17349.5  [8731.1 ; 29768.9] | 17189.3  [8760.6 ; 29093.5] | ↘ |

*test of trend or linear regression for values from 2010 to 2015; ↗ a significant increase is observed with a pvalue < 0.05; ↘ a significant decrease is observed with a pvalue < 0.05; ‘=’ no significant trend is observed ; SAPS II: simplified acute physiology score II; LOS: Length of stay; ICU: Intensive care unit

Table S7: Subgroup analyses of septic patients not admitted to ICU defined implicitly using ICD-10 or CCAM. Incidence, main characteristics, outcomes, costs and temporal trends from 2010 to 2015.

| ***Sepsis not admitted to ICU***  ***Implicit definition using CCAM or ICD-10*** | ***All*** | ***2010*** | ***2011*** | ***2012*** | ***2013*** | ***2014*** | ***2015*** | ***Trends**** |
| --- | --- | --- | --- | --- | --- | --- | --- | --- |
| *Cases* | 213756 | 30625 | 34675 | 37363 | 36606 | 37953 | 36534 |  |
| *Incidence per 100.000 (age-standardized)* |  | 59 | 66 | 70 | 68 | 69 | 66 | ↗ |
| *Deaths per 100.000 (age-standardized)* |  | 14 | 16 | 18 | 18 | 20 | 21 | ↗ |
| *Case fatality (age standardized)* |  | 24 | 25 | 26 | 27 | 28 | 32 | ↗ |
| *1st admission (between 2010 and 2015)* | 117589 (55) | 24848 (81.1) | 22882 (66) | 21233 (56.8) | 18229 (49.8) | 16674 (43.9) | 13723 (37.6) | ↘ |
| *Number of admissions, median (IQR)* | 1 [1 ; 2] | 1 [1 ; 1] | 1 [1 ; 2] | 1 [1 ; 2] | 2 [1 ; 2] | 2 [1 ; 3] | 2 [1 ; 3] | ↗ |
| *Readmission (during the year following the last admission)* | 79658 (37.3) | 9967 (32.5) | 11793 (34) | 13555 (36.3) | 13890 (37.9) | 15093 (39.8) | 15360 (42) | ↗ |
| *Baseline characteristics* |  |  |  |  |  |  |  |  |
| *Age in years, median (IQR)* | 77 [64 ; 85] | 76 [62 ; 84] | 76 [63 ; 84] | 77 [63 ; 85] | 77 [64 ; 85] | 77 [64 ; 85] | 78 [65 ; 86] | ↗ |
| *Female gender* | 91382 (42.8) | 13081 (42.7) | 14979 (43.2) | 15927 (42.6) | 15448 (42.2) | 16073 (42.3) | 15874 (43.4) | = |
| *Charlson score, median (IQR)* | 2 [1 ; 4] | 2 [1 ; 4] | 2 [1 ; 4] | 2 [1 ; 4] | 2 [1 ; 4] | 2 [1 ; 4] | 2 [1 ; 4] | ↗ |
| Severity of the patient illness |  |  |  |  |  |  |  |  |
| *Cardio vascular organ failure* | 125929 (58.9) | 17808 (58.1) | 20320 (58.6) | 21609 (57.8) | 21181 (57.9) | 22292 (58.7) | 22719 (62.2) | ↗ |
| *Number of organ failure, median (IQR)* | 1 [1 ; 2] | 1 [1 ; 2] | 1 [1 ; 2] | 1 [1 ; 2] | 1 [1 ; 2] | 1 [1 ; 2] | 2 [1 ; 2] | ↗ |
| Outcome |  |  |  |  |  |  |  |  |
| *Hospital LOS(days), median (IQR)* | 9 [4 ; 17] | 9 [3 ; 17] | 9 [3 ; 17] | 9 [4 ; 17] | 9 [4 ; 17] | 9 [4 ; 17] | 9 [4 ; 17] | ↗ |
| *Discharge to home* | 138550 (64.8) | 20581 (67.2) | 23262 (67.1) | 24632 (65.9) | 23785 (65) | 24338 (64.1) | 21952 (60.1) | ↘ |
| *Hospital mortality* | 59368 (27.8) | 7522 (24.6) | 8847 (25.5) | 9861 (26.4) | 10115 (27.6) | 10995 (29) | 12028 (32.9) | ↗ |
| *Cost (€), median (IQR)* | 4635.3  [2505.7 ; 6844.1] | 4553.5  [2195.7 ; 6778.4] | 4525.7  [2327.7 ; 6524.2] | 4534.7  [2456.4 ; 6555.6] | 4652.7  [2602.1 ; 6800.6] | 4678.8  [2725.5 ; 7113.1] | 4678.8  [2646.3 ; 7071.4] | ↗ |

*test of trend or linear regression for values from 2010 to 2015; ↗ a significant increase is observed with a pvalue < 0.05; ↘ a significant decrease is observed with a pvalue < 0.05; ‘=’ no significant trend is observed ; SAPS II: simplified acute physiology score II; LOS: Length of stay; ICU: Intensive care unit

Table S8: Subgroup analyses of the septic shock patients with septicemia defined implicitly using ICD-10 or CCAM. Main characteristics and outcomes and temporal trends from 2010 to 2015.

| ***Septic shock with septicemia***  ***Implicit definition using CCAM or ICD-10*** | ***All*** | ***2010*** | ***2011*** | ***2012*** | ***2013*** | ***2014*** | ***2015*** | *Trends** |
| --- | --- | --- | --- | --- | --- | --- | --- | --- |
| *Cases* | 113722 | 21122 | 18808 | 17986 | 16488 | 17350 | 21968 |  |
| *Incidence per 100.000 (age-standardized)* |  | 41 | 36 | 34 | 30 | 31 | 39 | = |
| *Deaths per 100.000 (age-standardized)* |  | 19 | 16 | 15 | 13 | 13 | 16 | ↘ |
| *Case fatality (age standardized)* |  | 47 | 45 | 44 | 44 | 43 | 42 | ↘ |
| *1st admission (between 2010 and 2015)* | 110981 (97.6) | 20915 (99) | 18403 (97.8) | 17529 (97.5) | 16017 (97.1) | 16832 (97) | 21285 (96.9) | ↘ |
| *Number of admissions, median (IQR)* | 1 [1 ; 1] | 1 [1 ; 1] | 1 [1 ; 1] | 1 [1 ; 1] | 1 [1 ; 1] | 1 [1 ; 1] | 1 [1 ; 1] | = |
| *Readmission (during the year following the last admission)* | 2387 (2.1) | 493 (2.3) | 405 (2.2) | 358 (2) | 333 (2) | 357 (2.1) | 441 (2) | ↘ |
| *Characteristics of the patient* |  |  |  |  |  |  |  |  |
| *Age in years, median (IQR)* | 68 [58 ; 78] | 68 [57 ; 78] | 68 [57 ; 78] | 68 [57 ; 78] | 68 [58 ; 78] | 68 [58 ; 78] | 68 [59 ; 78] | ↗ |
| *Female gender* | 41786 (36.7) | 8004 (37.9) | 6993 (37.2) | 6548 (36.4) | 5992 (36.3) | 6258 (36.1) | 7991 (36.4) | ↘ |
| *Charlson score, median (IQR)* | 3 [1 ; 5] | 2 [1 ; 5] | 3 [1 ; 5] | 3 [1 ; 5] | 3 [1 ; 5] | 3 [1 ; 5] | 3 [1 ; 5] | ↗ |
| Severity of the patient illness |  |  |  |  |  |  |  |  |
| *Number of organ failure, median (IQR)* | 4 [3 ; 5] | 4 [3 ; 5] | 4 [3 ; 5] | 4 [3 ; 5] | 4 [3 ; 5] | 4 [3 ; 5] | 4 [3 ; 5] | ↘ |
| *SAPS II score, median (IQR)(miss=13279)* | 53 [40 ; 68] | 54 [41 ; 70] | 53 [40 ; 69] | 53 [40 ; 68] | 53 [40 ; 69] | 52 [40 ; 68] | 52 [40 ; 68] | ↘ |
| *ICU admission* | 103097 (90.7) | 19413 (91.9) | 17109 (91) | 16269 (90.5) | 14924 (90.5) | 15683 (90.4) | 19699 (89.7) | ↘ |
| Outcome |  |  |  |  |  |  |  |  |
| *Hospital LOS(days), median (IQR)* | 27 [12 ; 51] | 25 [11 ; 49] | 27 [12 ; 51] | 27 [12 ; 52] | 27 [12 ; 52] | 28 [13 ; 53] | 26 [12 ; 49] | ↗ |
| *ICU LOS(days), median (IQR)(miss=23917)* | 11 [4 ; 24] | 10 [4 ; 23] | 11 [4 ; 25] | 11 [4 ; 24] | 11 [4 ; 24] | 11 [4 ; 25] | 10 [4 ; 23] | = |
| *Difference between hospital and ICU stay, median (IQR)* | 14 [4 ; 32] | 12 [2 ; 29] | 14 [3 ; 31] | 14 [4 ; 32] | 16 [5 ; 34] | 15 [4 ; 33] | 15 [4 ; 31] | ↗ |
| *Discharge to home* | 47673 (41.9) | 8162 (38.6) | 7723 (41.1) | 7628 (42.4) | 6878 (41.7) | 7535 (43.4) | 9747 (44.4) | ↗ |
| *Hospital mortality* | 50930 (44.8) | 10065 (47.7) | 8581 (45.6) | 8074 (44.9) | 7406 (44.9) | 7530 (43.4) | 9274 (42.2) | ↘ |
| *Cost (€), median (IQR)* | 21291.2  [10278.2 ; 36988.7] | 21182.2  [10228.5 ; 35908.5] | 21239.2  [10181.7 ; 37291.5] | 21427.3  [10206.1 ; 37673.7] | 21897  [10508.4 ; 37942.8] | 21926.9  [10460.8 ; 38412.2] | 20375.8  [9830.8 ; 35448.8] | = |

*test of trend or linear regression for values from 2010 to 2015; ↗ a significant increase is observed with a pvalue < 0.05; ↘ a significant decrease is observed with a pvalue < 0.05; ‘=’ no significant trend is observed ; SAPS II: simplified acute physiology score II; LOS: Length of stay; ICU: Intensive care unit

Table S9: Subgroup analyses of the septic shock patients admitted to ICU defined implicitly using ICD-10 or CCAM. Main characteristics and outcomes and temporal trends from 2010 to 2015.

| ***Septic shock admitted to ICU***  ***Implicit definition using CCAM or ICD-10*** | ***All*** | ***2010*** | ***2011*** | ***2012*** | ***2013*** | ***2014*** | ***2015*** | *Trends** |
| --- | --- | --- | --- | --- | --- | --- | --- | --- |
| *Cases* | 421026 | 59482 | 64631 | 68728 | 70067 | 77300 | 80818 |  |
| *Incidence per 100.000 (age-standardized)* |  | 116 | 124 | 130 | 130 | 142 | 146 | ↗ |
| *Deaths per 100.000 (age-standardized)* |  | 51 | 53 | 55 | 55 | 58 | 59 | ↗ |
| *Case fatality (age standardized)* |  | 44 | 43 | 42 | 42 | 41 | 40 | ↘ |
| *1st admission (between 2010 and 2015)* | 402543 (95.6) | 58602 (98.5) | 62687 (97) | 65981 (96) | 66618 (95.1) | 72923 (94.3) | 75732 (93.7) | ↘ |
| *Number of admissions, median (IQR)* | 1 [1 ; 1] | 1 [1 ; 1] | 1 [1 ; 1] | 1 [1 ; 1] | 1 [1 ; 1] | 1 [1 ; 1] | 1 [1 ; 1] | = |
| *Readmission (during the year following the last admission)* | 14214 (3.4) | 1909 (3.2) | 1944 (3) | 2181 (3.2) | 2408 (3.4) | 2728 (3.5) | 3044 (3.8) | ↗ |
| *Characteristics of the patient* |  |  |  |  |  |  |  |  |
| *Age in years, median (IQR)* | 68 [57 ; 78] | 68 [56 ; 78] | 68 [57 ; 78] | 68 [57 ; 78] | 68 [58 ; 79] | 68 [58 ; 79] | 68 [58 ; 78] | ↗ |
| *Female gender* | 157334 (37.4) | 22338 (37.6) | 24096 (37.3) | 25853 (37.6) | 26205 (37.4) | 28865 (37.3) | 29977 (37.1) | = |
| *Charlson score, median (IQR)* | 2 [1 ; 4] | 2 [1 ; 4] | 2 [1 ; 4] | 2 [1 ; 4] | 2 [1 ; 4] | 2 [1 ; 5] | 2 [1 ; 4] | ↗ |
| Severity of the patient illness |  |  |  |  |  |  |  |  |
| *Number of organ failure, median (IQR)* | 4 [3 ; 5] | 4 [3 ; 5] | 4 [3 ; 5] | 4 [3 ; 5] | 4 [3 ; 5] | 4 [3 ; 5] | 4 [3 ; 5] | = |
| *SAPS II score, median (IQR)(miss=16301)* | 51 [38 ; 67] | 51 [39 ; 67] | 51 [38 ; 67] | 51 [39 ; 67] | 51 [38 ; 67] | 51 [38 ; 67] | 52 [39 ; 68] | = |
| Outcome |  |  |  |  |  |  |  |  |
| *Hospital LOS(days), median (IQR)* | 22 [10 ; 42] | 23 [10 ; 44] | 23 [10 ; 43] | 22 [10 ; 43] | 23 [10 ; 42] | 22 [10 ; 42] | 21 [10 ; 40] | ↘ |
| *ICU LOS(days), median (IQR)(miss=63231)* | 8 [3 ; 18] | 9 [3 ; 20] | 9 [3 ; 19] | 8 [3 ; 19] | 8 [3 ; 19] | 8 [3 ; 18] | 8 [3 ; 17] | ↘ |
| *Difference between hospital and ICU stay, median (IQR)* | 12 [2 ; 26] | 11 [2 ; 26] | 11 [2 ; 26] | 12 [2 ; 26] | 13 [3 ; 27] | 12 [3 ; 26] | 11 [3 ; 25] | = |
| *Discharge to home* | 176271 (41.9) | 23533 (39.6) | 26374 (40.8) | 28558 (41.6) | 29213 (41.7) | 33552 (43.4) | 35041 (43.4) | ↗ |
| *Hospital mortality* | 178044 (42.3) | 26235 (44.1) | 28014 (43.3) | 29458 (42.9) | 29595 (42.2) | 31787 (41.1) | 32955 (40.8) | ↘ |
| *Cost (€), median (IQR)* | 19305.7  [10178.5 ; 32095.3] | 20151.9  [10424.8 ; 33465.5] | 19215.6  [9868.6 ; 32565.7] | 18916.5  [9776.8 ; 32015.4] | 19437.4  [10324.3 ; 32151] | 19289.9  [10297.4 ; 31853.4] | 18990.9  [10229.8 ; 30988.4] | ↘ |

*test of trend or linear regression for values from 2010 to 2015; ↗ a significant increase is observed with a pvalue < 0.05; ↘ a significant decrease is observed with a pvalue < 0.05; ‘=’ no significant trend is observed ; SAPS II: simplified acute physiology score II; LOS: Length of stay; ICU: Intensive care unit

Table S10: Subgroup analyses of the septic shock patients not admitted to ICU defined implicitly using ICD-10 or CCAM. Main characteristics and outcomes and temporal trends from 2010 to 2015.

| ***Septic shock not admitted to ICU***  ***Implicit definition using CCAM or ICD-10*** | ***All*** | ***2010*** | ***2011*** | ***2012*** | ***2013*** | ***2014*** | ***2015*** | *Trends** |
| --- | --- | --- | --- | --- | --- | --- | --- | --- |
| *Cases* | 69486 | 9611 | 11295 | 11945 | 11461 | 12214 | 12960 |  |
| *Incidence per 100.000 (age-standardized)* |  | 19 | 22 | 23 | 23 | 24 | 25 | ↗ |
| *Deaths per 100.000 (age-standardized)* |  | 11 | 13 | 14 | 14 | 15 | 16 | ↗ |
| *Case fatality (age standardized)* |  | 59 | 59 | 60 | 62 | 63 | 64 | ↗ |
| *1st admission (between 2010 and 2015)* | 65543 (94.3) | 9341 (97.2) | 10752 (95.2) | 11271 (94.4) | 10761 (93.9) | 11369 (93.1) | 12049 (93) | ↘ |
| *Number of admissions, median (IQR)* | 1 [1 ; 1] | 1 [1 ; 1] | 1 [1 ; 1] | 1 [1 ; 1] | 1 [1 ; 1] | 1 [1 ; 1] | 1 [1 ; 1] | = |
| *Readmission (during the year following the last admission)* | 3297 (4.7) | 386 (4) | 543 (4.8) | 596 (5) | 534 (4.7) | 625 (5.1) | 613 (4.7) | = |
| *Baseline characteristics* |  |  |  |  |  |  |  |  |
| *Age in years, median (IQR)* | 81 [70 ; 88] | 80 [68 ; 87] | 81 [68 ; 87] | 81 [69 ; 88] | 82 [70 ; 88] | 82 [70 ; 88] | 83 [71 ; 89] | ↗ |
| *Female gender* | 32684 (47) | 4570 (47.5) | 5262 (46.6) | 5517 (46.2) | 5466 (47.7) | 5663 (46.4) | 6206 (47.9) | = |
| *Charlson score, median (IQR)* | 2 [0 ; 3] | 2 [0 ; 3] | 2 [0 ; 3] | 2 [0 ; 3] | 2 [0 ; 4] | 2 [0 ; 4] | 2 [0 ; 3] | ↗ |
| *Severity of the patient illness* |  |  |  |  |  |  |  |  |
| *Number of organ failure, median (IQR)* | 2 [2 ; 3] | 2 [2 ; 3] | 2 [2 ; 3] | 2 [2 ; 3] | 2 [2 ; 3] | 2 [2 ; 3] | 2 [2 ; 3] | ↗ |
| Outcome |  |  |  |  |  |  |  |  |
| *Hospital LOS(days), median (IQR)* | 6 [1 ; 15] | 6 [1 ; 16] | 6 [1 ; 15] | 6 [1 ; 15] | 6 [1 ; 15] | 6 [1 ; 15] | 6 [1 ; 15] | ↘ |
| *Discharge to home* | 21255 (30.6) | 3240 (33.7) | 3788 (33.5) | 3827 (32) | 3271 (28.5) | 3518 (28.8) | 3611 (27.9) | ↘ |
| *Hospital mortality* | 43994 (63.3) | 5749 (59.8) | 6846 (60.6) | 7418 (62.1) | 7455 (65) | 7970 (65.3) | 8556 (66) | ↗ |
| *Cost (€), median (IQR)* | 4743.9  [891.4 ; 7272.3] | 4808.2  [896.9 ; 7638] | 4648.5  [891.4 ; 7063.4] | 4534.7  [873 ; 6801] | 4668.5  [828.9 ; 7218.4] | 4874.6  [822.5 ; 7560.7] | 4761.7  [822.5 ; 7397.9] | = |

*test of trend or linear regression for values from 2010 to 2015; ↗ a significant increase is observed with a pvalue < 0.05; ↘ a significant decrease is observed with a pvalue < 0.05; ‘=’ no significant trend is observed ; SAPS II: simplified acute physiology score II; LOS: Length of stay; ICU: Intensive care unit

Table S11: Identifications of factors associated with death - multivariate hierarchical logistic regression analyses with a random center effect - for the patients with sepsis and septic shock.

|  | Imp. Sepsis | | | Imp. Septic shock | | |
| --- | --- | --- | --- | --- | --- | --- |
|  | CCAM or ICD-10 | | | ICD-10 or CCAM | | |
| **Variables (No (%))** | **OR** | **OR** | **OR** | **OR** | **OR CI** | **Pvalue** |
| **Hospital level** |  |  |  |  |  | 0.01 |
| **Admission for sepsis level** |  |  |  |  |  |  |
| **Year of admission** |  |  |  |  |  |  |
| 2011 | 1.01 | [0.99 ; 1.03] |  | 1.00 | [0.98 ; 1.02] |  |
| 2012 | 1.00 | [0.98 ; 1.02] |  | 0.98 | [0.96 ; 1] |  |
| 2013 | 1.00 | [0.98 ; 1.02] |  | 0.97 | [0.94 ; 0.99] |  |
| 2014 | 1.00 | [0.98 ; 1.02] |  | 0.95 | [0.93 ; 0.97] |  |
| 2015 | 1.03 | [1.01 ; 1.05] |  | 0.94 | [0.92 ; 0.96] |  |
| 2010 |  |  | <0.01 |  |  | <0.01 |
| **Reason of admission** |  |  |  |  |  |  |
| Medical versus surgical | 1.95 | [1.93 ; 1.98] | <0.01 | 2.08 | [2.04 ; 2.11] | <0.01 |
| Readmission | 1.10 | [1.09 ; 1.12] | <0.01 | 0.83 | [0.8 ; 0.86] | <0.01 |
| **Origin of the patient** |  |  |  |  |  |  |
| From home | 0.83 | [0.81 ; 0.84] | <0.01 | 0.89 | [0.87 ; 0.91] | <0.01 |
| **Service of admission** |  |  |  |  |  |  |
| ICU | 0.69 | [0.67 ; 0.7] | <0.01 | 0.30 | [0.3 ; 0.31] | <0.01 |
| Emergency department | 0.89 | [0.87 ; 0.9] | <0.01 | 0.77 | [0.76 ; 0.78] | <0.01 |
| **Baseline characteristics** |  |  |  |  |  |  |
| Age (y) | 1.04 | [1.04 ; 1.04] | <0.01 | 1.04 | [1.04 ; 1.04] | <0.01 |
| Sex | 1.01 | [1 ; 1.03] | 0.02 | 1.00 | [0.98 ; 1.01] | 0.79 |
| **Comorbidities** |  |  |  |  |  |  |
| Myocardial infarction | 0.79 | [0.77 ; 0.8] | <0.01 | 0.79 | [0.77 ; 0.8] | <0.01 |
| Congestive heart failure | 0.99 | [0.97 ; 1] | 0.05 | 0.91 | [0.9 ; 0.93] | <0.01 |
| Peri-vascular disease | 0.98 | [0.96 ; 1] | 0.02 | 0.96 | [0.94 ; 0.98] | <0.01 |
| Cerebro-vascular disease | 1.24 | [1.22 ; 1.27] | <0.01 | 1.30 | [1.27 ; 1.32] | <0.01 |
| Dementia | 0.89 | [0.86 ; 0.91] | <0.01 | 0.80 | [0.78 ; 0.83] | <0.01 |
| Chronic pulmonary disease | 0.71 | [0.69 ; 0.72] | <0.01 | 0.75 | [0.73 ; 0.76] | <0.01 |
| Rheumatoid disease | 0.89 | [0.86 ; 0.93] | <0.01 | 0.93 | [0.89 ; 0.97] | <0.01 |
| Peptic ulcer disease | 0.74 | [0.72 ; 0.76] | <0.01 | 0.77 | [0.75 ; 0.79] | <0.01 |
| Mild liver disease | 1.76 | [1.72 ; 1.8] | <0.01 | 1.80 | [1.76 ; 1.85] | <0.01 |
| Diabetes without chronic complication | 0.71 | [0.7 ; 0.73] | <0.01 | 0.70 | [0.69 ; 0.71] | <0.01 |
| Diabetes with chronic complications | 0.89 | [0.87 ; 0.9] | <0.01 | 0.89 | [0.87 ; 0.91] | <0.01 |
| Hemiplegia or paraplegia | 0.84 | [0.82 ; 0.86] | <0.01 | 0.79 | [0.76 ; 0.81] | <0.01 |
| Renal disease | 0.90 | [0.88 ; 0.91] | <0.01 | 0.94 | [0.92 ; 0.96] | <0.01 |
| Any malignancy | 1.40 | [1.38 ; 1.43] | <0.01 | 1.29 | [1.27 ; 1.31] | <0.01 |
| Moderate or severe liver disease | 1.26 | [1.22 ; 1.29] | <0.01 | 1.25 | [1.21 ; 1.29] | <0.01 |
| Metastatic solid tumor | 2.23 | [2.17 ; 2.28] | <0.01 | 1.81 | [1.76 ; 1.86] | <0.01 |
| HIV | 1.55 | [1.45 ; 1.66] | <0.01 | 1.60 | [1.48 ; 1.72] | <0.01 |
| **Illness severity** |  |  |  |  |  |  |
| Cardiovascular organ failure | 10.81 | [10.53 ; 11.09] | <0.01 |  |  |  |
| Hematological organ failure | 1.44 | [1.42 ; 1.47] | <0.01 | 1.46 | [1.43 ; 1.48] | <0.01 |
| Neurological organ failure | 1.54 | [1.52 ; 1.56] | <0.01 | 1.42 | [1.4 ; 1.44] | <0.01 |
| Renal organ failure | 1.85 | [1.83 ; 1.88] | <0.01 | 2.07 | [2.05 ; 2.1] | <0.01 |
| Respiratory organ failure | 2.81 | [2.77 ; 2.86] | <0.01 | 2.55 | [2.5 ; 2.61] | <0.01 |
| SAPS II |  |  |  |  |  |  |
| **Pathogen identified** | | | |  |  |  |
| Gram negative bacilli |  |  |  | 0.97 | [0.95 ; 0.99] | <0.01 |
| Gram positive cocci |  |  |  | 0.97 | [0.95 ; 0.99] | <0.01 |
| Fungal infection | 1.05 | [1.03 ; 1.07] | <0.01 | 1.09 | [1.06 ; 1.11] | <0.01 |
| Viral infection | 0.74 | [0.72 ; 0.76] | <0.01 | 0.78 | [0.75 ; 0.8] | <0.01 |
| No pathogen identified | 1.44 | [1.43 ; 1.46] | <0.01 | 1.54 | [1.5 ; 1.58] | <0.01 |
| **Site of infection** |  |  |  |  |  |  |
| Pulmonary tract | 0.85 | [0.84 ; 0.86] | <0.01 | 0.83 | [0.82 ; 0.84] | <0.01 |
| Intra-abdominal | 0.94 | [0.93 ; 0.96] | <0.01 | 0.94 | [0.92 ; 0.95] | <0.01 |
| Urinary tract | 0.47 | [0.46 ; 0.47] | <0.01 | 0.41 | [0.4 ; 0.42] | <0.01 |
| Septicemia | 1.18 | [1.16 ; 1.2] | <0.01 | 1.06 | [1.04 ; 1.08] | <0.01 |

SAPS II: simplified acute physiology score II; OR: Odd ratio; OR CI: Odd Ratio Confidence Interval

**Variables tested in univariate analysis:** Private versus public institutions, medical versus surgical motif of admission, admission from home, admission in ICU or intermediate care facilities, Emergency department, baseline characteristics including age, sex, comorbidities such as myocardial infarction, congestive heart failure, peri vascular disease, cerebro-vascular disease, dementia, chronic pulmonary disease, rheumatic disease, peptic ulcer disease, mild liver disease, diabetes without chronic complication, diabetes with chronic complications, hemiplegia or paraplegia, renal disease, any malignancy, moderate or severe liver disease, metastatic solid tumor, AIDS/HIV, severity of the patient including hematological, neurological, renal and respiratory organ failures, type of pathogen with Gram negative bacilli, Gram positive cocci, fungal infection, viral infection, no pathogen identifications, site of infection with pulmonary, abdominal, urinary infections and septicemia.


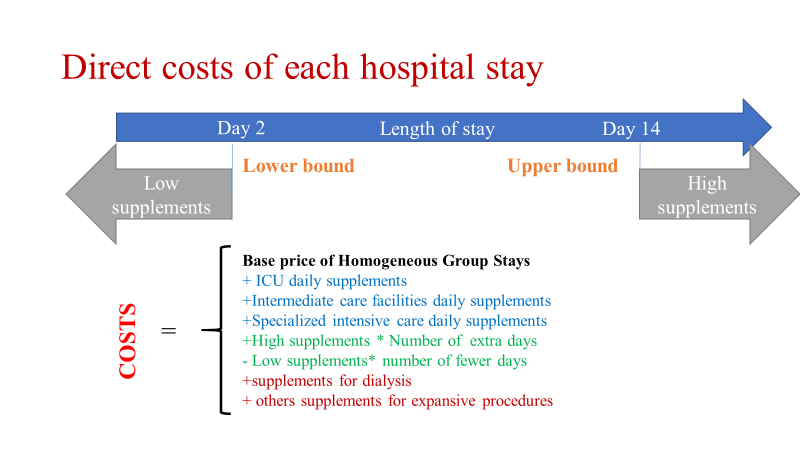


Figure S1: Direct costs of each hospital stay

ICU: Intensive care unit; ICD-10: International Statistical Classification of Diseases, 10th Edition. CCAM: “*classification commune des actes médicaux*”

ICD coding allows the classification of each hospital stay in a homogenous group stay (HGS) which provides the base price of the hospital stay. HGS are never directly related to sepsis or septic shock but only to a specific infection (i. e. pneumonia, endocarditis, …). However, depending on the severity of the infection which can be defined by a code for sepsis or septic shock or the SAPS II score, the HGS is categorized into four levels of increasing prices.

CCAM procedure codes are necessary to define ICU daily supplements which are added to the base price of a hospital stay. This coding system is known to be representative of the actual practices and likely more rigorous and systematic than ICD-10 coding.

The direct costs of each hospital stay were estimated using the base price of Homogeneous Group Stays and the rates of daily supplements based on extra days and special procedures. In the specific case of sepsis, a diagnostic code for sepsis or septic shock or a highest SAPS II score might help to trigger a Homogenous Group stay of higher value and procedure codes were necessary to get an ICU/intermediate care facilities daily supplement.


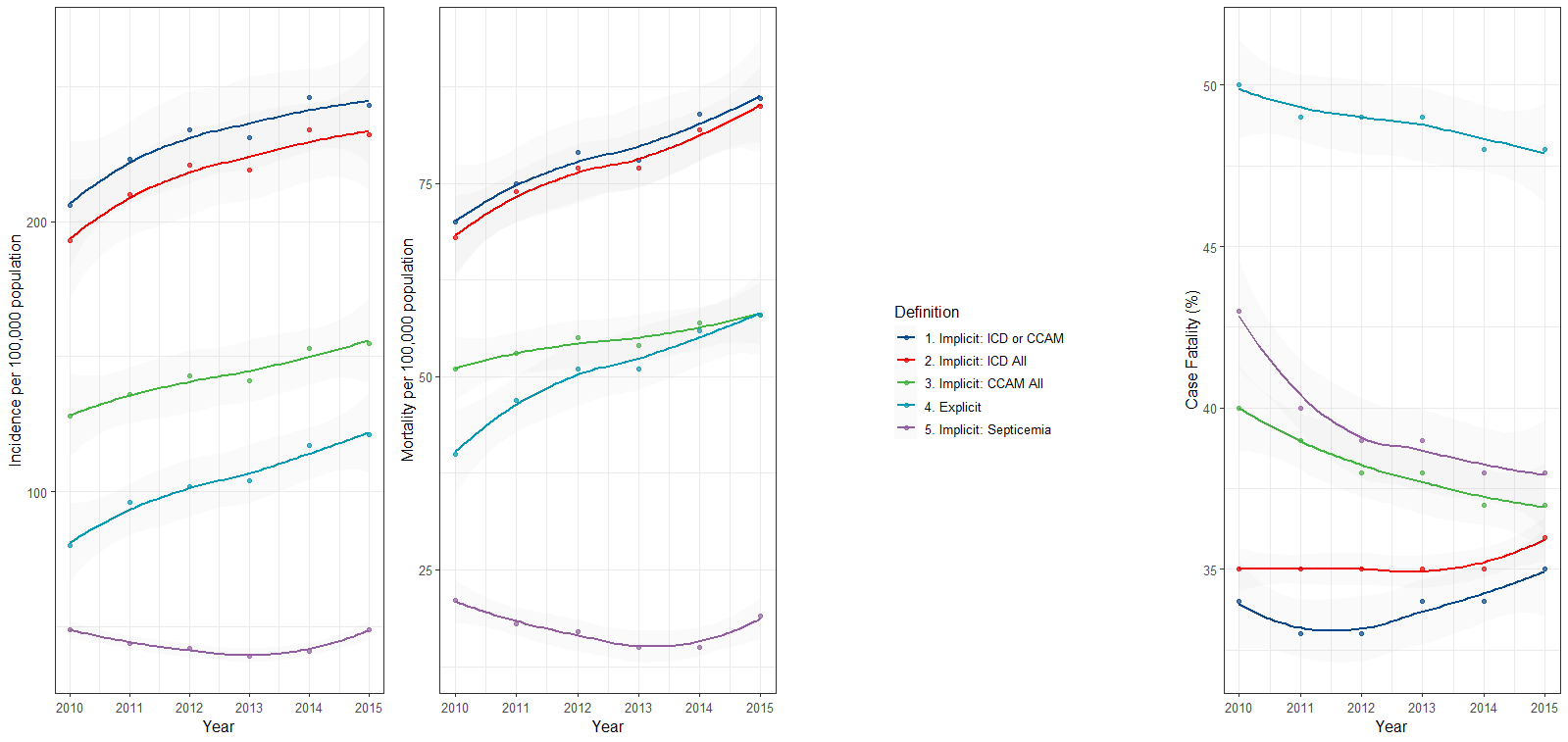


Figure S2: Standardized incidence, mortality and case fatality for patients with sepsis depending on explicit or implicit definitions of sepsis.

CD-10: International Statistical Classification of Diseases, 10th Edition. CCAM: “*classification commune des actes médicaux”*
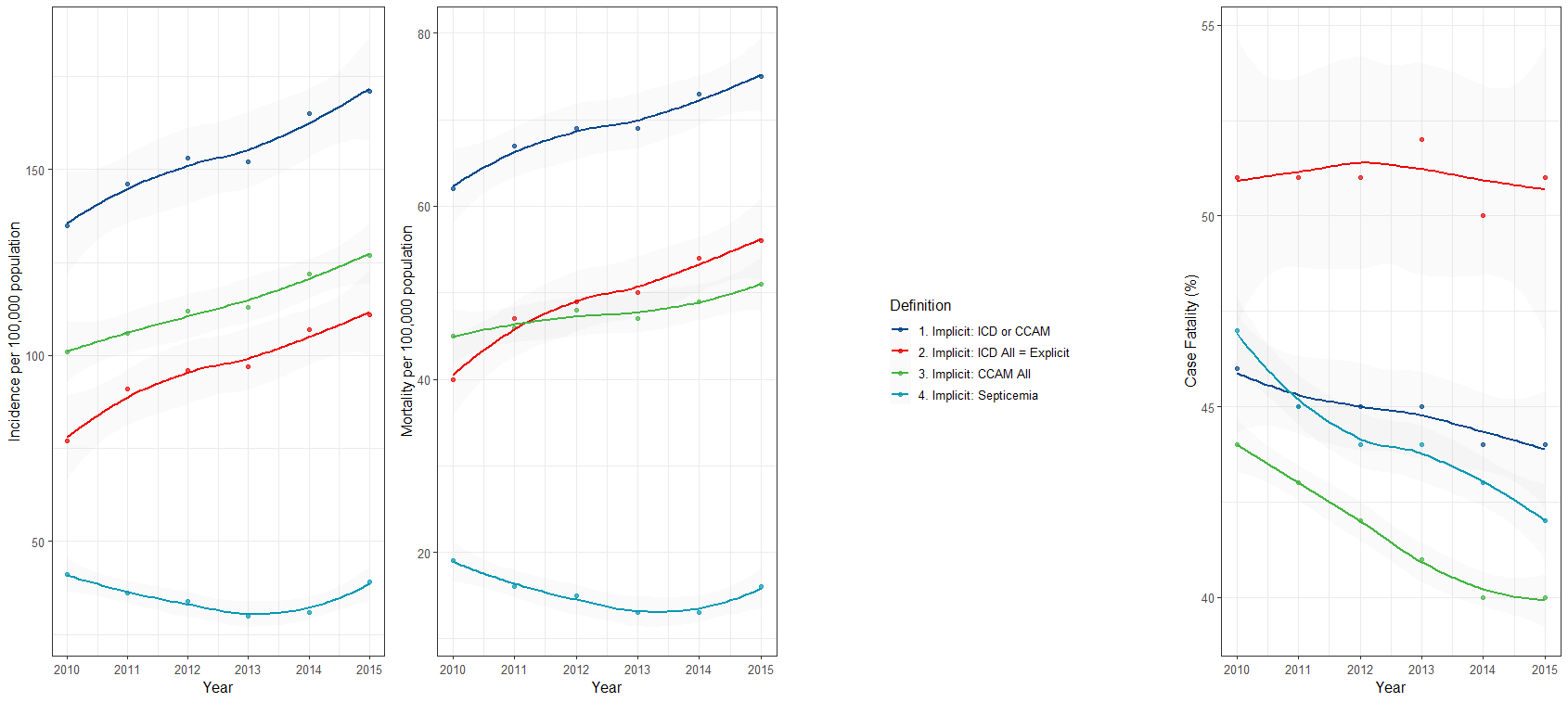


Figure S3: Standardized incidence, mortality and case fatality for patients with septic shock depending on explicit or implicit definitions of septic shock.

ICD-10: International Statistical Classification of Diseases, 10th Edition. CCAM: “*classification commune des actes médicaux*

**REFERENCES**

1. Angus DC, Linde-Zwirble WT, Lidicker J, Clermont G, Carcillo J, Pinsky MR. Epidemiology of severe sepsis in the United States: analysis of incidence, outcome, and associated costs of care. Crit Care Med. 2001;29:1303–10.

2. Martin GS, Mannino DM, Eaton S, Moss M. The epidemiology of sepsis in the United States from 1979 through 2000. N Engl J Med. 2003;348:1546–54.

3. Wang HE, Shapiro NI, Angus DC, Yealy DM. National estimates of severe sepsis in United States emergency departments: Crit Care Med. 2007;35:1928–36.

4. Dombrovskiy VY, Martin AA, Sunderram J, Paz HL. Rapid increase in hospitalization and mortality rates for severe sepsis in the United States: A trend analysis from 1993 to 2003*: Crit Care Med. 2007;35:1244–50.

5. Yang Y, Yang KS, Hsann YM, Lim V, Ong BC. The effect of comorbidity and age on hospital mortality and length of stay in patients with sepsis. J Crit Care. 2010;25:398–405.

6. Kumar G, Kumar N, Taneja A, Kaleekal T, Tarima S, McGinley E, et al. Nationwide trends of severe sepsis in the 21st century (2000-2007). Chest. 2011;140:1223–31.

7. Lagu T, Rothberg MB, Shieh M-S, Pekow PS, Steingrub JS, Lindenauer PK. What is the best method for estimating the burden of severe sepsis in the United States? J Crit Care. 2012;27:414.e1-414.e9.

8. Lagu T, Rothberg MB, Shieh M-S, Pekow PS, Steingrub JS, Lindenauer PK. Hospitalizations, costs, and outcomes of severe sepsis in the United States 2003 to 2007: Crit Care Med. 2012;40:754–61.

9. Bouza C, López-Cuadrado T, Saz-Parkinson Z, Amate-Blanco JM. Epidemiology and recent trends of severe sepsis in Spain: a nationwide population-based analysis (2006-2011). BMC Infect Dis. 2014;14:3863.

10. Ani C, Farshidpanah S, Bellinghausen Stewart A, Nguyen HB. Variations in Organism-Specific Severe Sepsis Mortality in the United States: 1999–2008*. Crit Care Med. 2015;43:65–77.

11. Stoller J, Halpin L, Weis M, Aplin B, Qu W, Georgescu C, et al. Epidemiology of severe sepsis: 2008-2012. J Crit Care. 2016;31:58–62.

12. Gohil SK, Cao C, Phelan M, Tjoa T, Rhee C, Platt R, et al. Impact of Policies on the Rise in Sepsis Incidence, 2000–2010. Clin Infect Dis. 2016;62:695–703.

13. Kadri SS, Rhee C, Strich JR, Morales MK, Hohmann S, Menchaca J, et al. Estimating Ten-Year Trends in Septic Shock Incidence and Mortality in United States Academic Medical Centers Using Clinical Data. Chest. 2017;151:278–85.

14. Rhee C, Kadri S, Huang SS, Murphy MV, Li L, Platt R, et al. Objective Sepsis Surveillance Using Electronic Clinical Data. Infect Control Hosp Epidemiol. 2016;37:163–71.

15. Elfeky S, Golabi P, Otgonsuren M, Djurkovic S, Schmidt ME, Younossi ZM. The epidemiologic characteristics, temporal trends, predictors of death, and discharge disposition in patients with a diagnosis of sepsis: A cross-sectional retrospective cohort study. J Crit Care. 2017;39:48–55.

16. Yébenes JC, Ruiz-Rodriguez JC, Ferrer R, Clèries M, Bosch A, Lorencio C, et al. Epidemiology of sepsis in Catalonia: analysis of incidence and outcomes in a European setting. Ann Intensive Care. 2017;7:19.

17. Rhee C, Dantes R, Epstein L, Murphy DJ, Seymour CW, Iwashyna TJ, et al. Incidence and Trends of Sepsis in US Hospitals Using Clinical vs Claims Data, 2009-2014. JAMA. 2017;318:1241.

18. Lee C-C, Yo C-H, Lee M-TG, Tsai K-C, Lee S-H, Chen Y-S, et al. Adult sepsis - A nationwide study of trends and outcomes in a population of 23 million people. J Infect. 2017;75:409–19.

19. Fleischmann-Struzek C, Mikolajetz A, Schwarzkopf D, Cohen J, Hartog CS, Pletz M, et al. Challenges in assessing the burden of sepsis and understanding the inequalities of sepsis outcomes between National Health Systems: secular trends in sepsis and infection incidence and mortality in Germany. Intensive Care Med. 2018;

20. Quan H, Sundararajan V, Halfon P, Fong A, Burnand B, Luthi J-C, et al. Coding algorithms for defining comorbidities in ICD-9-CM and ICD-10 administrative data. Med Care. 2005;43:1130–9.
